# TOTEM: A low-cost solar-powered aeroponic system for vertical agriculture

**DOI:** 10.1016/j.ohx.2026.e00838

**Published:** 2026-09-05

**Authors:** Fernando D. Von Borstel, Juan Francisco Villa-Medina, Alejandra Nieto-Garibay, Joaquín Gutiérrez

**Affiliations:** Engineering Group, Organic Agriculture, Centro de Investigaciones Biológicas del Noroeste, S.C., La Paz, Mexico

**Keywords:** Energy autonomy, Urban farming, Modular platform

## Abstract

Soil degradation, freshwater scarcity, and urbanization are increasing the demand for resource-efficient food production systems. Controlled environment agriculture (CEA), particularly aeroponics, enables efficient crop production with reduced water and nutrient use; however, most existing systems rely on grid electricity and involve high implementation costs.

This work presents TOTEM, an open-source, solar-powered vertical aeroponic system implemented for energy-autonomous soilless agriculture. Developed for universal replication, the design allows for modification or scaling using widely available materials. The system integrates a modular stacked architecture comprising growth, reservoir, pumping, filtration, control, and power units. A microcontroller-based embedded controller manages irrigation cycles and monitors chamber temperature and nutrient solution level, whereas a photovoltaic module enables fully off-grid operation.

The system was built to support up to 24 plants within a compact footprint (0.078 m^3^) and validated using basil (*Ocimum basilicum*) under two successive harvest cycles. Results demonstrated stable irrigation performance, uniform nutrient distribution, and reliable operation on solar power. Biomass production confirmed the system capability to sustain plant growth under continuous operation.

TOTEM provides a scalable, low-cost platform for vertical farming with applications in urban food production, research, and education, particularly in resource-limited or off-grid environments.

## Graphical abstract

**Figure:**
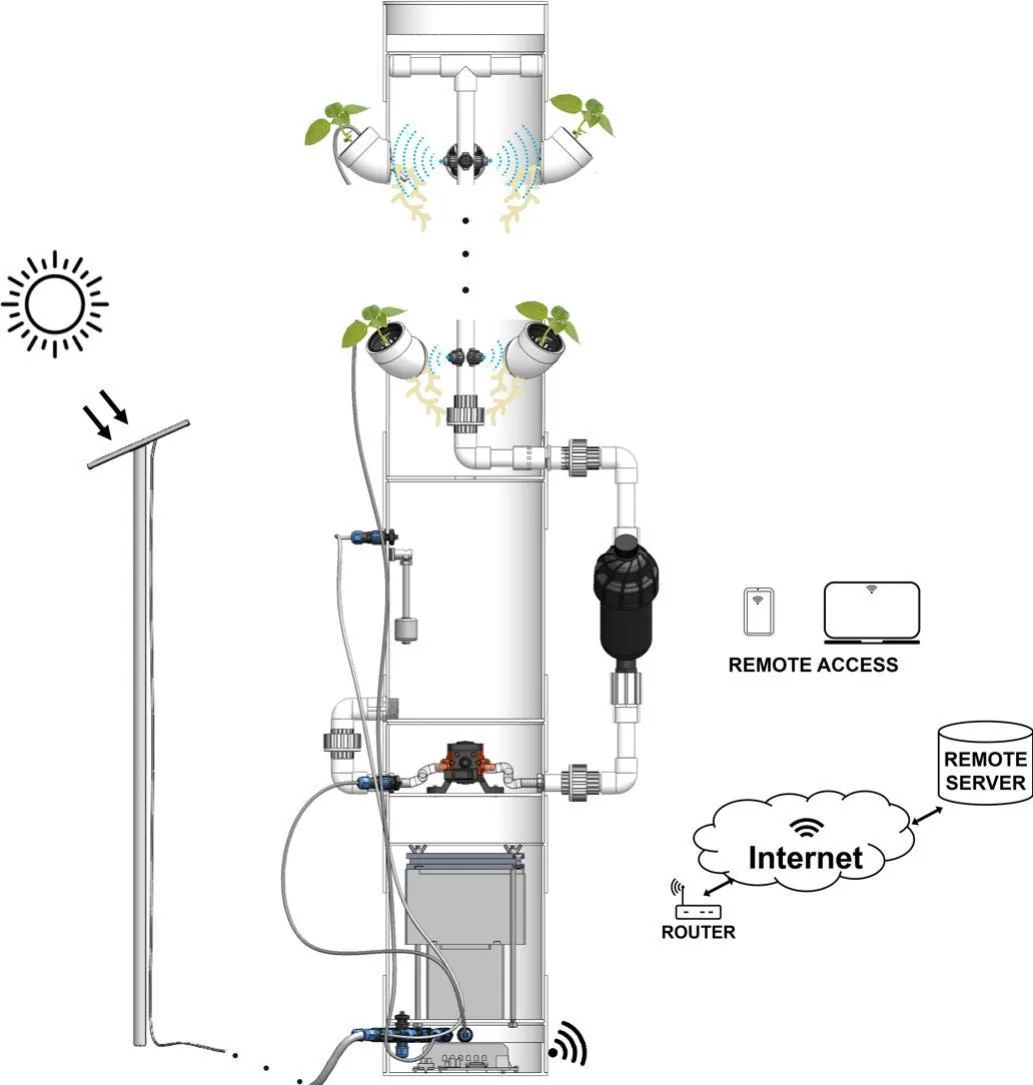

**Table:** 

Hardware name	TOTEM Solar Aeroponic System
Subject area	• Engineering • Environmental and Agricultural Sciences • Open-source and educational tools
Hardware type	• Electrical engineering and computer science
Closest commercial analog	Vertical aeroponic tower systems
Open-source license	GNU General Public License v3, CC by 4.0 and CERN OHL v2-S
Cost of hardware	$720 USD
Source file repository	https://doi.org/10.17632/nsbtxzhyt6.1

## 1 Hardware in context

Freshwater scarcity, soil degradation, and rapid urbanization are increasing pressure on agricultural systems. Irrigated agriculture accounts for approximately 70% of global freshwater withdrawals, while arable-land availability continues to decline. These constraints are driving the adoption of controlled-environment agriculture (CEA) to increase productivity while reducing resource consumption. The Food and Agriculture Organization estimates that global agricultural production will need to increase by about 50% by 2050 relative to 2012 [1]. Within CEA, urban farming uses horizontal or vertical growing arrangements in multistory buildings to maximize space and limited resources for urban food production [2], [3].

Soilless cultivation includes hydroponics, aquaponics, and aeroponics [4], [5], [6]. Hydroponics uses a nutrient solution as the primary growing medium, plants are supported in cultivation units without soil, with shoots exposed to air and roots in the nutrient solution [5]. Hydroponics is generally practiced using deep water culture (DWC) systems, which employ nutrient-filled rectangular beds and floating rafts to support plants, and nutrient film technique (NFT) systems that circulate a thin nutrient-solution layer through grow channels from inlet to outlet [7]. Aquaponics combines hydroponic crop production with aquaculture in a symbiotic system. Aeroponics, which is also categorized as a hydroponic technique [8], suspends roots in a closed chamber and sprays or mists them with nutrient solution [3].

Among these techniques, aeroponics offers the highest potential for water-use efficiency, with reported reductions of 90–98% relative to soil-based cultivation, while enhancing root oxygenation and nutrient uptake [9], [10]. Additional reported benefits include precise moisture control, reduced exposure to soil-borne diseases, space-efficient cultivation, and potential reductions in operational costs [11], [12]; efficient and economical advantages over other techniques have also been reported [13]. These characteristics support plant research, sustainable agriculture, and space-related applications [14], including food production under microgravity [15].

Aeroponic performance depends on careful management and monitoring of environmental conditions. Sensor networks, the Internet of Things (IoT), artificial intelligence (AI), and embedded microcontroller platforms are used to monitor or regulate irrigation, lighting, nutrient-solution conditions, and greenhouse climate [16], [17], [18], [19], [20], [21], [22], [23]. Across hydroponic and aeroponic applications, these technologies have been associated with more efficient use of agricultural inputs and increased yields [24].

Aeroponic systems also depend predominantly on continuous electrical power [25]. An outage can rapidly compromise plant survival through root desiccation and nutrient deprivation, while commercial systems are often costly, proprietary, and difficult to adapt for research or resource-limited settings.

TOTEM addresses these constraints through an open-source, low-cost, modular, solar-powered aeroponic system for vertical agriculture. Its compact architecture integrates irrigation, sensing, and renewable energy for autonomous operation using widely available materials. Detailed hardware documentation, modularity, and off-grid capability are intended to expand access to aeroponic technology for urban farming, research, and education, particularly in resource-limited environments.

### 1.1 Literature review

Hydroponic prototypes illustrate several embedded monitoring and control architectures. Michael et al. [26] compared NFT and flood-and-drain energy consumption using an Arduino Mega 2560 and ESP8266 NodeMCU system linked to an IoT cloud platform. The system monitored electrical conductivity (EC), pH, water level, and nutrient solution temperature and controlled motorized valves and a nutrient solution pump. Sneineh and Shabaneh [27] used an ESP32 in a DWC system to acquire temperature, water level, pH, and total dissolved solids (TDS), process the measurements, and activate water, nutrient, or phosphoric-acid pumps as required; monitoring and control were available through an IoT smartphone application. For passive Kratky cultivation, Kushawaha et al. [28] proposed an Arduino Mega 2560 master–slave architecture that monitored water level and flow, temperature, humidity, soil moisture, and nitrogen, phosphorus, and potassium (NPK), and controlled a water pump.

Aeroponic monitoring and control systems based on wireless sensing are reviewed by Lakhiar et al. [13]. Reported implementations include an Arduino Mega 2560 platform for measuring temperature, flow, pH, and nutrient-solution EC and controlling greenhouse conditions [22], and a Lattepanda mini PC for automating irrigation, lighting, and climate control [29]. Giotopoulos et al. [30] developed an IoT-based greenhouse architecture in which a sensor cluster connected to a Raspberry Pi 4 monitored and controlled temperature, relative humidity, light intensity, ventilation, and CO2 in real time. Rajendiran et al. [31] used machine learning to predict yield from sensor measurements of nutrient-solution pH, EC, turbidity, and TDS; ambient humidity, temperature, and photosynthetically active radiation (PAR); and lettuce phenotypic metrics, including canopy growth rate and final yield (g/plant).

The cited hydroponic and aeroponic systems are experimental prototypes developed to propose and validate specific monitoring, control, or prediction approaches. Their reports provide limited detail on the aeroponic hardware and omit 3D model files for subsequent machining or additive manufacturing.

Energy use is a dominant component of CEA greenhouse production costs, accounting for 10–40% of total production costs [2], [32]. The cited implementations omit a sustainable energy source for cultivation irrigation and environmental parameter monitoring. Together, the limited fabrication documentation and dependence on continuous power define the hardware and energy gaps addressed by TOTEM.

### 1.2 Related commercial systems

Several commercial aeroponic systems with varying capabilities are currently available for both home and greenhouse applications (Table 1). The Tower Garden FLEX growing system [33], developed by Tower Garden (Collierville, TN, USA), is a vertical aeroponic platform featuring a 20-plant tower that can be expanded using an extension kit. The system is constructed from UV-stabilized, food-grade plastic and is distributed as a complete starter kit including all necessary components.

**Table 1:** Comparison of vertical aeroponic systems.

Feature	Tower garden FLEX [33]	Pro aerotower [34]	Tower farm [35]	TOTEM
Plants	28	48	28	24
Power	Grid	Grid	Grid	Solar-powered
Energy autonomy	No	No	No	Yes
Irrigation setting	*in situ*	*in situ*	*in situ*	Remote (GUI)
Irrigation	Low-pressure	High-pressure	Low-pressure	Low-pressure
Monitoring	*in situ*	*in situ*	*in situ*	Remote (GUI)
Filtration	Not specified	Not specified	Not specified	Mesh filter
Material	Food-grade plastic	Food-grade PP	Food-grade PC/ABS	PVC
Open-source	No	No	No	Yes
Cost (USD)	765	799	5250	720
Footprint (m3)	0.74	0.4	0.74	0.078

*Minimum order of 10 towers; GUI, graphical user interface.

The Pro AeroTower system from Nutraponics (Germantown, MD, USA) is a high-pressure vertical aeroponic system designed to support up to 48 plants [34]. It employs high-pressure misting nozzles that produce droplets smaller than 50 µm, thereby enhancing oxygen availability to plant roots. The structure is manufactured from FDA-approved, food-grade polypropylene (PP) and is also provided as a complete system.

Similarly, Agrotonomy (Sedona, AZ, USA) offers the Aeroponics Tower Farm system [35], which supports up to 28 plants. This system is constructed from UV-stabilized, BPA-free, food-grade PC/ABS materials and includes all required components within a proprietary starter kit.

Despite their advanced design and commercial availability, these systems present several limitations. None of the reviewed platforms incorporate solar-powered operation, relying instead on continuous grid electricity. In addition, they generally lack integrated filtration mechanisms, such as mesh filters, to prevent clogging of irrigation lines and misting nozzles. Furthermore, their cost and structural configuration often result in larger system footprints and reduced accessibility for low-resource or off-grid applications.

## 2 Hardware description

The TOTEM is a modular, vertically integrated aeroponic platform that combines plant cultivation, nutrient delivery, automated control, and autonomous energy supply within a compact structure. The prototype was developed at the Centro de Investigaciones Biológicas del Noroeste (CIBNOR) as an open hardware system intended for reproducible and adaptable applications.

### 2.1 System architecture

**Fig. 1:**
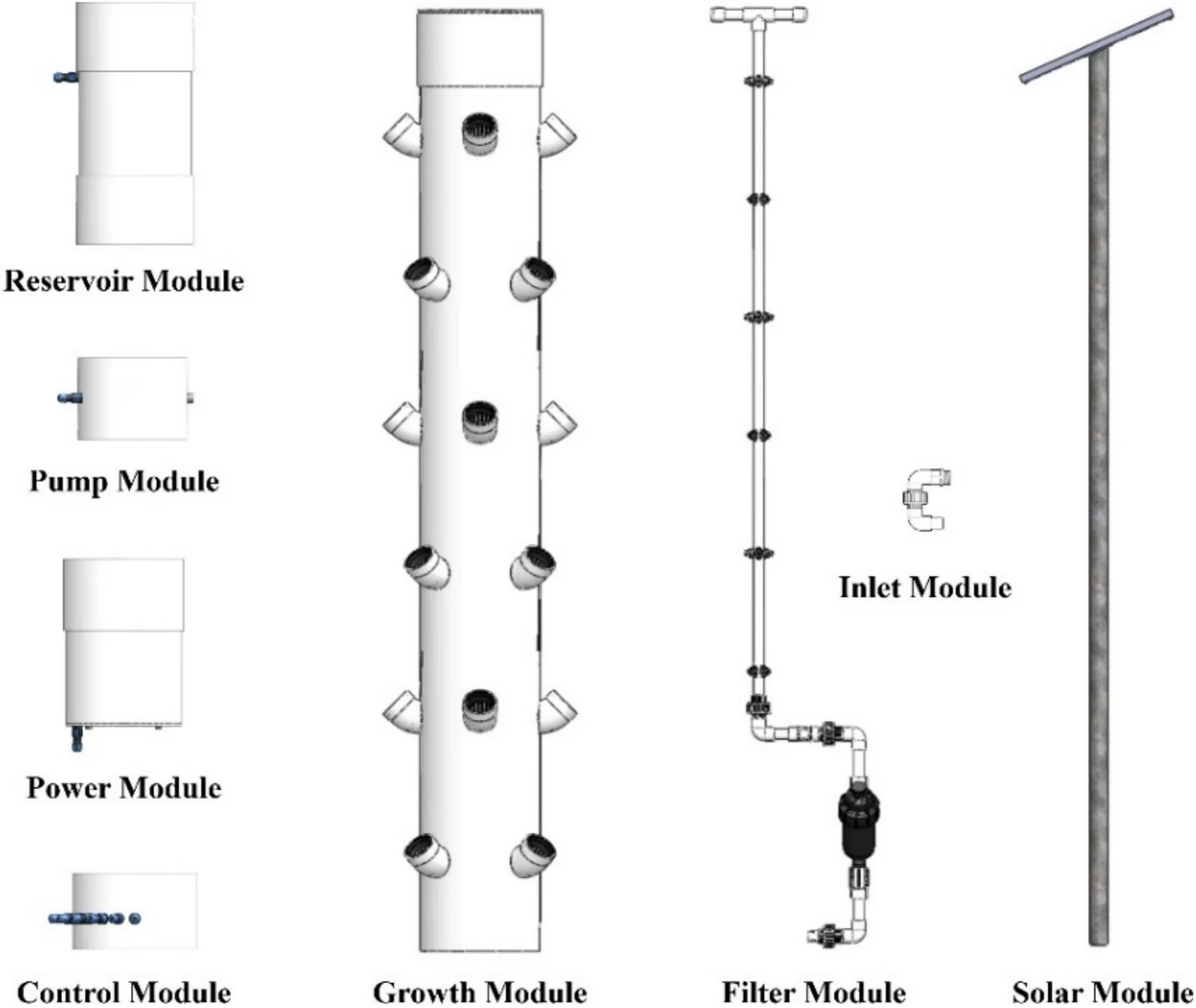
Modules of the low-cost solar aeroponic system: TOTEM.

The system is configured as a vertically stacked cylindrical structure assembled from PVC components with a diameter of 0.20 m. Its architecture comprises eight mechanically coupled and functionally interconnected modules: growth, reservoir, pump, filter, inlet–drainage, control, power, and solar modules (Fig. 1). Together, these modules support nutrient solution recirculation, environmental monitoring, irrigation control, wireless communication, and off-grid operation.

### 2.2 Growth module

The Growth module consists of a 1.5 m-high PVC tower with capacity for up to 24 plants. The planting sites are arranged in a staggered circular configuration using angled PVC elbow fittings, which optimize the available vertical space and improve light distribution among plants. Each plant is held in a net cup, with its roots suspended inside the tower chamber and directly exposed to the aeroponic mist. This configuration facilitates root oxygenation and nutrient uptake. The modular structure also permits adjustment of tower height and plant density according to cultivation requirements.

A vertical distribution pipe extends through the internal chamber of the Growth module. Multiple misting nozzles installed along this pipe atomize the pressurized nutrient solution into fine droplets directed toward the suspended roots.

### 2.3 Reservoir module

The Reservoir module stores the nutrient solution supplied to the hydraulic circuit and collects the excess solution returning from the Growth module. A water-level sensor installed in the reservoir provides input to the control system for solution-level monitoring. Returning the drained solution to the reservoir supports closed-loop recirculation and reduces water and nutrient losses.

### 2.4 Pump module

The Pump module uses a 12 V DC diaphragm pump to withdraw nutrient solution from the reservoir and provide the pressure required for filtration and atomization. Pump activation is controlled through an electrical relay connected to the microcontroller, allowing operation according to the programmed irrigation schedule or commands received through the graphical user interface.

### 2.5 Filter module

The Filter module contains a mesh filtration unit installed in the nutrient-delivery path. The filter captures suspended particles before the solution reaches the distribution pipe and misting nozzles, thereby reducing the risk of nozzle obstruction and maintaining hydraulic flow through the irrigation circuit.

### 2.6 Inlet module

The Inlet module provides hydraulic interfaces among the Reservoir, Rump, Filter, and Growth modules. The inlet path directs the pressurized and filtered nutrient solution toward the vertical distribution pipe. After atomization, excess solution flows downward through the tower by gravity and returns to the reservoir through the drainage path, completing the recirculation loop.

### 2.7 Control module

The Control module contains the microcontroller and associated electronic interfaces responsible for irrigation scheduling, sensor-data acquisition, pump activation, and communication. The microcontroller receives measurements from a digital temperature sensor located within the growth chamber and a water-level sensor installed in the reservoir. It also controls the pump through an electrical relay.

Wi-Fi connectivity enables real-time monitoring and remote operation through a graphical user interface. The interface allows users to adjust irrigation parameters, activate the pump, and monitor system variables and operating status.

### 2.8 Power module

The Power module contains two 12 V rechargeable batteries that store the electrical energy supplied by the photovoltaic system. A charge controller regulates battery charging and power delivery to the pump and control electronics. Energy storage allows irrigation and monitoring functions to continue during periods of variable solar irradiance.

### 2.9 Solar module

The Solar module consists of a photovoltaic panel mounted on a structural mast to increase exposure to incident sunlight. The panel supplies electrical energy to the charge controller and battery bank, enabling autonomous operation independent of the electrical grid.

### 2.10 Integrated operation

During operation, the pump draws nutrient solution from the reservoir and delivers it through the mesh filter to the vertical distribution pipe inside the growth tower. The misting nozzles atomize the solution and direct it toward the suspended plant roots. Excess nutrient solution subsequently drains by gravity into the reservoir for reuse. The microcontroller coordinates pump activation, acquires temperature and water-level measurements, and exchanges operating information with the graphical user interface through Wi-Fi.

The mechanical, hydraulic, control, and energy interfaces form a compact aeroponic platform capable of closed-loop nutrient recirculation and off-grid operation. Standardized PVC components and commercially available electronic devices facilitate fabrication, replacement, and replication. The modular architecture also supports adaptation of the tower height, plant capacity, irrigation configuration, and energy subsystem to different cultivation requirements.

## 3 Design files summary

### 3.1 Totem-level design files

The modular components of the TOTEM system are listed in Table 2 and illustrated in Fig. 1.

**Table 2:** Design files for the TOTEM system.

Design filename	File type	Open-source license	Location of the file
module_control	CAD Assembly (STEP)	CERN OHL v2-S	Available in file repository
module_power	CAD Assembly (STEP)	CERN OHL v2-S	Available in file repository
module_pump	CAD Assembly (STEP)	CERN OHL v2-S	Available in file repository
module_reservoir	CAD Assembly (STEP)	CERN OHL v2-S	Available in file repository
module_growth	CAD Assembly (STEP)	CERN OHL v2-S	Available in file repository
module_filter	CAD Assembly (STEP)	CERN OHL v2-S	Available in file repository
module_inlet	CAD Assembly (STEP)	CERN OHL v2-S	Available in file repository
module_solar	CAD Assembly (STEP)	CERN OHL v2-S	Available in file repository

Description:

1. module_control: This assembly houses the electronics and controller core.
2. module_power: This file represents the energy storage module for energy autonomy.
3. module_pump: This assembly includes the water pump and internal fluid routing for nutrient delivery.
4. module_reservoir: This design file includes the nutrient solution container and the level sensor.
5. module_growth: This assembly defines the main cultivation tower, including the plant cup units.
6. module_filter: An assembly managing water filtration and the vertical misting line for the aeroponic system.
7. module_inlet: This file contains the intake and drainage plumbing interface for the system.
8. module_solar: This design module provides solar power generation for system autonomy.

### 3.2 Control module

**Fig. 2:**
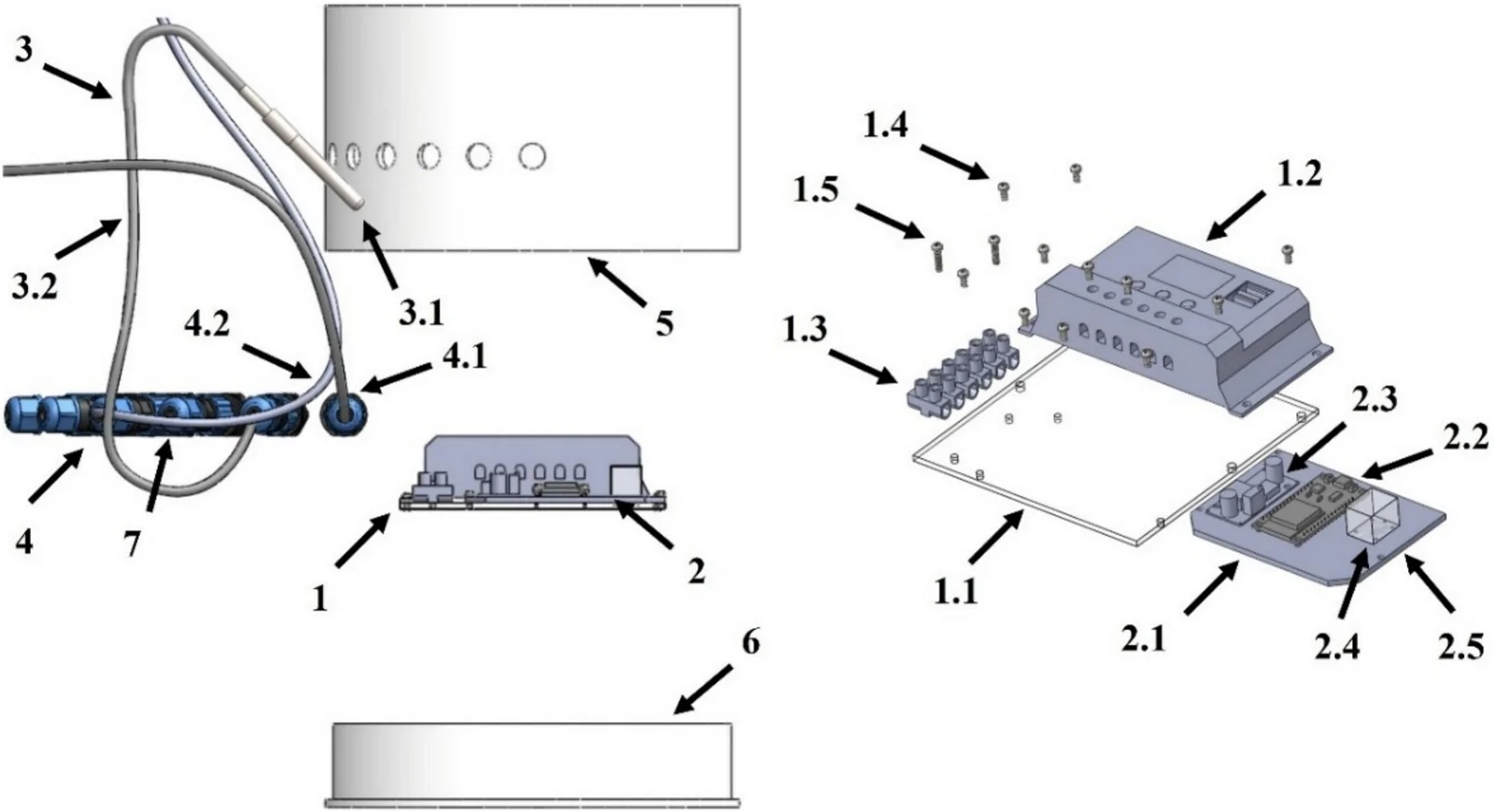
Control module components: unit_control (1), unit_pcb (2), unit_temperature (3), connector_sp13 (4), couple_control (5), cap_control (6), base_control (1.1), controller (1.2), terminal (1.3), screw_3x6 (1.4), screw_3x12 (1.5), pcb_control (2.1), esp32_wroom (2.2), regulator_5V (2.3), relay_RAS1220M (2.4), optocoupler_FOD852 (2.5), sensor_temperature (3.1), cable_sensor (3.2), cable_pump (4.1), cable_level (4.2), and connector_switch (7).

The constituent parts of the Control module are listed in Table 3 and illustrated in Fig. 2.

**Table 3:** Design files for the Control module.

Design file name	File type	Open-source license	Location of the file
unit_control	CAD Assembly (STEP)	CERN OHL v2-S	Available in file repository
unit_pcb	CAD Assembly (STEP)	CERN OHL v2-S	Available in file repository
unit_temperature	CAD Assembly (STEP)	CERN OHL v2-S	Available in file repository
connector_sp13	CAD Assembly (STEP)	CERN OHL v2-S	Available in file repository
couple_control	CAD Part (STEP)	CERN OHL v2-S	Available in file repository
cap_control	CAD Part (STEP)	CERN OHL v2-S	Available in file repository
base_control	CAD Part (STEP)	CERN OHL v2-S	Available in file repository
controller	CAD Part (STEP)	CERN OHL v2-S	Available in file repository
terminal	CAD Part (STEP)	CERN OHL v2-S	Available in file repository
screw_3x6	CAD Part (STEP)	CERN OHL v2-S	Available in file repository
screw_3x12	CAD Part (STEP)	CERN OHL v2-S	Available in file repository
pcb_control	CAD Part (STEP)	CERN OHL v2-S	Available in file repository
esp32_wroom	CAD Part (STEP)	CERN OHL v2-S	Available in file repository
regulator_5V	CAD Part (STEP)	CERN OHL v2-S	Available in file repository
optocoupler_FOD852	CAD Part (STEP)	CERN OHL v2-S	Available in file repository
relay_RAS1220M	CAD Part (STEP)	CERN OHL v2-S	Available in file repository
sensor_temperature	CAD Part (STEP)	CERN OHL v2-S	Available in file repository
cable_sensor	CAD Part (STEP)	CERN OHL v2-S	Available in file repository
cable_pump	CAD Part (STEP)	CERN OHL v2-S	Available in file repository
cable_level	CAD Part (STEP)	CERN OHL v2-S	Available in file repository

Description:

1. unit_control: Internal sub-assembly grouping base, controller, terminal block, and PCB.
2. unit_pcb: Electronic sub-assembly consisting of the custom PCB and its mounted components.
3. unit_temperature: Sub-assembly unit with the temperature sensor and its cable.
4. connector_sp13: SP13 connectors used for external power and data interfaces. A SP13 connector used as a switch (connector_switch); the male plug is wired to function as a closed jumper.
5. couple_control: External 8 PVC coupler as housing for the Control module.
6. cap_control: Standard 8 PVC cap used to seal the bottom of the control housing.
7. base_control: Acrylic base plate that provides the mounting surface for internal components.
8. controller: Solar charge controller 40A MPPT with Liquid Crystal Display (LCD) screen.
9. terminal: Wire barrier terminal strip block 6 × 2.
10. screw_3x6: Screws 3 mm × 6 mm (M3 × 6).
11. screw_3x12: Screws 3 mm × 12 mm (M3 × 12).
12. pcb_control: In-house designed printed circuit board layout for electronics.
13. esp32_wroom: ESP32 WROOM microcontroller model used as the central processing unit.
14. regulator_5V: 5 V LM2596 voltage regulator board used for power management on the PCB.
15. optocoupler_FOD852: FOD852 optocoupler used to activate the relay.
16. relay_RAS1220M: Relay component used for switching the irrigation pump.
17. sensor_temperature: Waterproof 1-Wire DS18B20 digital temperature sensor.
18. cable_sensor: 22 AWG 3 conductors.
19. cable_pump: 22 AWG electrical 2-wire parallel.
20. cable_level: 22 AWG electrical 2-wire parallel.

### 3.3 Power module

**Fig. 3:**
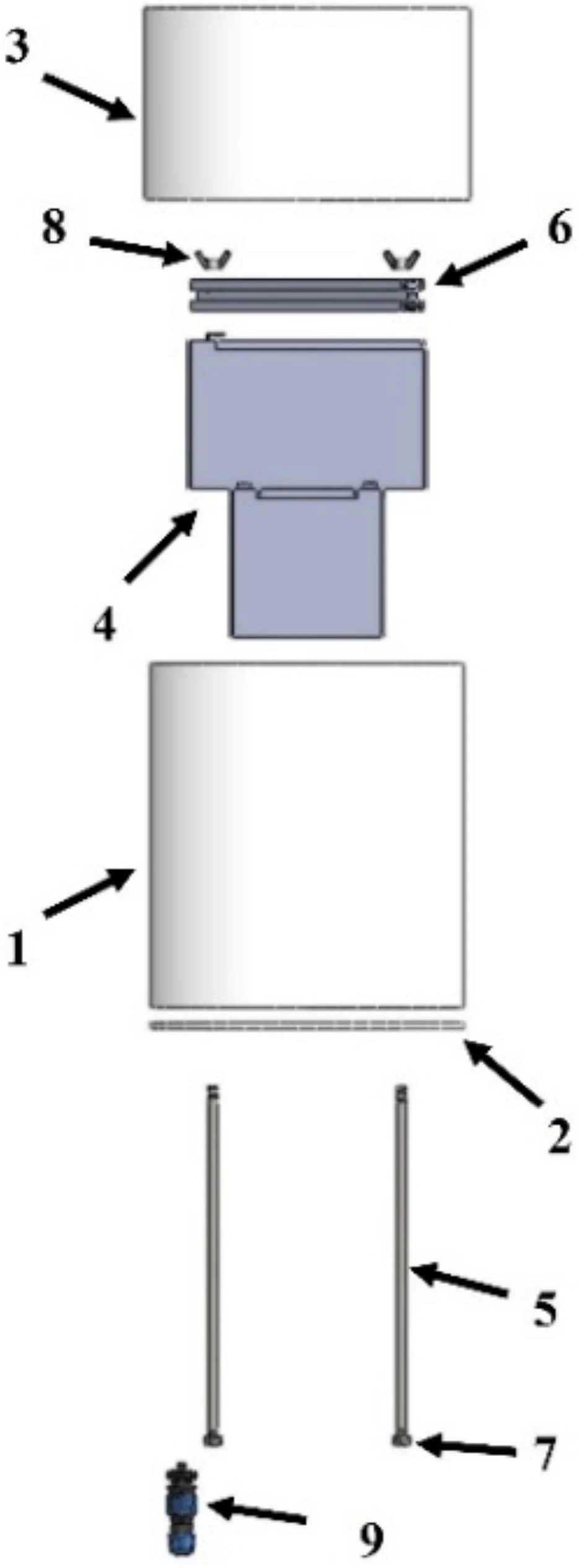
Power module components: shell_power (1), cap_power (2), couple_power (3), battery_12V (4), pole_battery (5), bar_battery (6), nut_M6 (7), wingnut_M6 (8), and connector_sp13 (9).

The constituent parts of the Power module are listed in Table 4 and illustrated in Fig. 3.

**Table 4:** Design files for the Power module.

Design filename	File type	Open-source license	Location of the file
shell_power	CAD Part (STEP)	CERN OHL v2-S	Available in file repository
cap_power	CAD Part (STEP)	CERN OHL v2-S	Available in file repository
couple_power	CAD Part (STEP)	CERN OHL v2-S	Available in file repository
battery_12V	CAD Part (STEP)	CERN OHL v2-S	Available in file repository
pole_battery	CAD Part (STEP)	CERN OHL v2-S	Available in file repository
bar_battery	CAD Part (STEP)	CERN OHL v2-S	Available in file repository
nut_M6	CAD Part (STEP)	CERN OHL v2-S	Available in file repository
wingnut_M6	CAD Part (STEP)	CERN OHL v2-S	Available in file repository
connector_sp13	CAD Assembly (STEP)	CERN OHL v2-S	Available in file repository

Descriptions:

1. shell_power: 8 PVC SCH40 pipe section (220 mm) as housing component for the power section.
2. cap_power: 8 PVC cap machined to hold the battery units within the module.
3. couple_power_200: 8 PVC coupler used as the exterior casing for the Power module.
4. battery_12V: 12 V-12Ah rechargeable battery unit used in a parallel configuration.
5. pole_battery: 1/4 steel threaded pole used to vertically secure the battery stack.
6. bar_battery: Aluminum 20 × 20 bar that locks the batteries in place when fastened.
7. nut_M6: 1/4–20 stainless steel hex nuts.
8. wingnut_M6: 1/4–20 stainless steel wing nuts.
9. connector_sp13: SP13 connector utilized for establishing external power.

### 3.4 Pump module

**Fig. 4:**
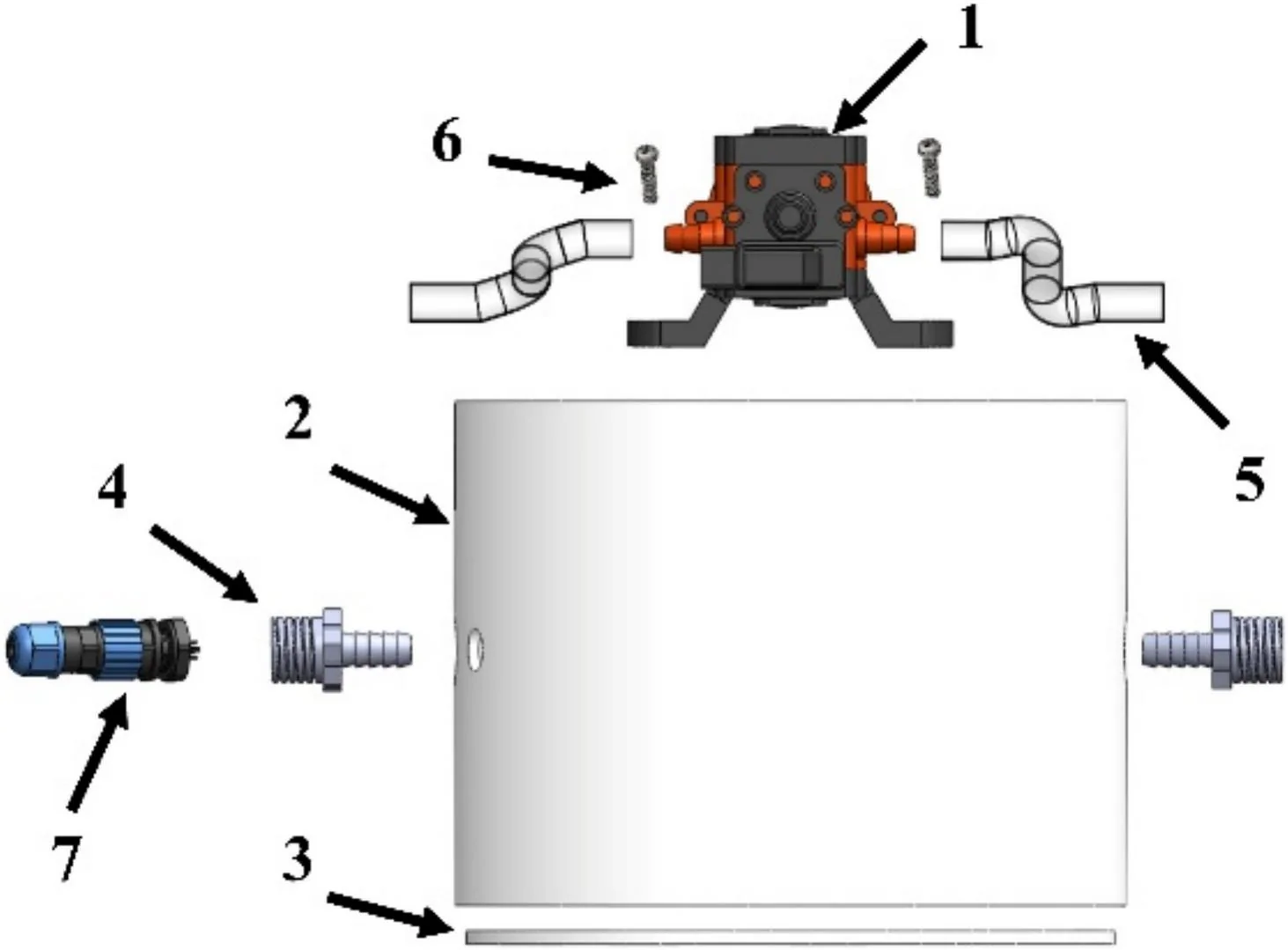
Pump module components: water_pump (1), shell_pump (2), cap_pump (3), adapter_LFA385 (4), hose (5), screw_3x12 (6), and connector_sp13 (7).

The constituent parts of the Pump module are listed in Table 5 and illustrated in Fig. 4.

**Table 5:** Design files for the Pump module.

Design filename	File type	Open-source license	Location of the file
water_pump	CAD Assembly (STEP)	CERN OHL v2-S	Available in file repository
shell_pump	CAD Part (STEP)	CERN OHL v2-S	Available in file repository
cap_pump	CAD Part (STEP)	CERN OHL v2-S	Available in file repository
adapter_LFA385	CAD Part (STEP)	CERN OHL v2-S	Available in file repository
hose	CAD Part (STEP)	CERN OHL v2-S	Available in file repository
screw_3x12	CAD Part (STEP)	CERN OHL v2-S	Available in file repository
connector_sp13	CAD Assembly (STEP)	CERN OHL v2-S	Available in file repository

Description:

1. water_pump: Electric pump moves nutrient solution through the system.
2. shell_pump_200: 8 PVC SCH40 pipe section (150 mm) as housing for the irrigation delivery module.
3. cap_pump: 8 PVC cap machined to support the pump within the housing.
4. adapter_95_127: 1/2 NPT brass adapter used for fluid connections at the pump interface.
5. hose: 1/2 hose section used for routing fluid within the Pump module.
6. screw_3x12: Screws 3 mm × 12 mm (M3 × 12).
7. connector_sp13: SP13 connector utilized to provide external power to the water pump.

### 3.5 Reservoir module

**Fig. 5:**
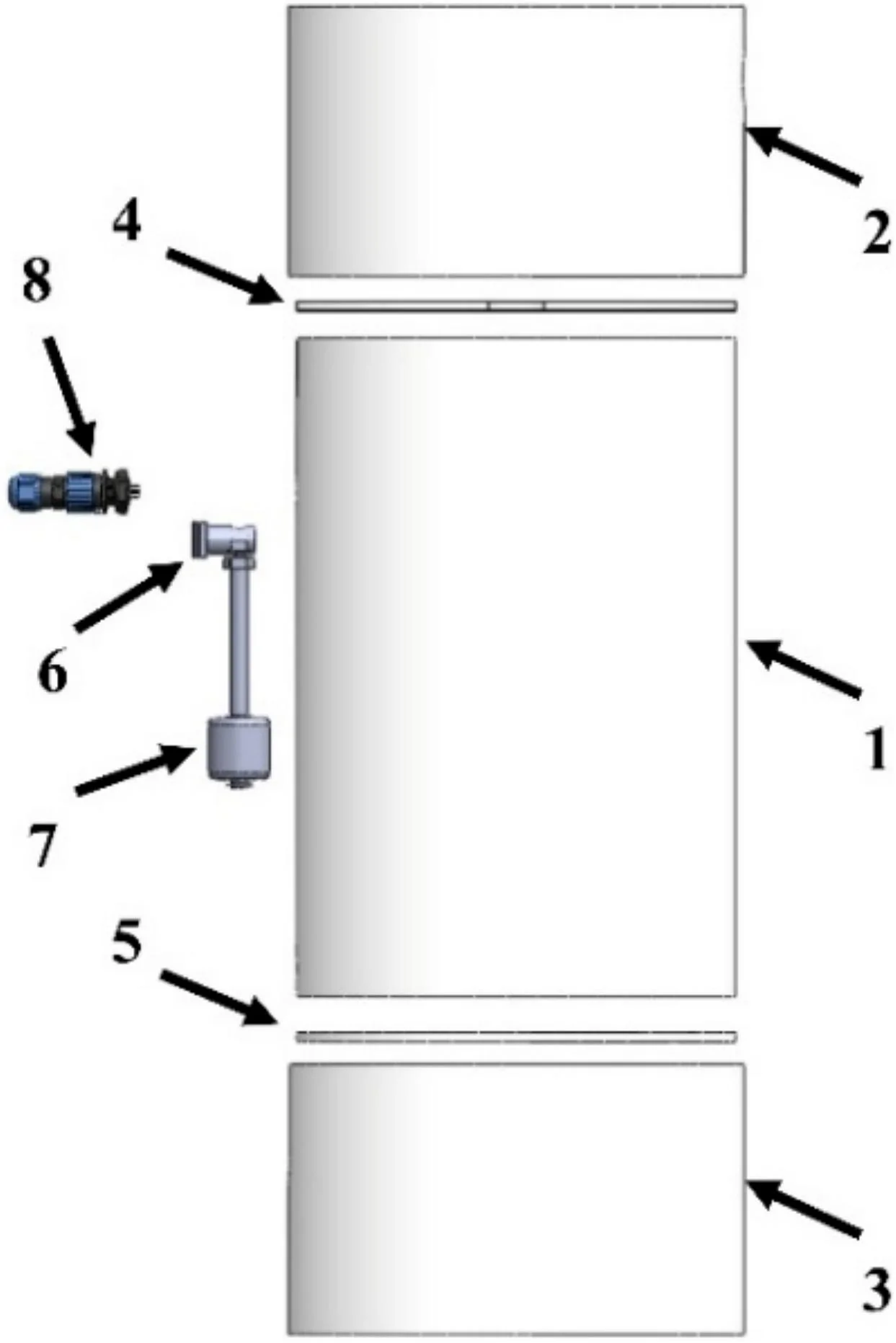
Reservoir module components: shell_reservoir (1), couple_reservoir_top (2), couple_reservoir_bottom (3), cap_reservoir_top (4), cap_reservoir_bottom (5), base_sensor (6), sensor_water_level (7), and connector_sp13 (8).

The constituent parts of the Reservoir module are listed in Table 6 and illustrated in Fig. 5.

**Table 6:** Design files for the Reservoir module.

Design filename	File type	Open-source license	Location of the file
shell_reservoir	CAD Part (STEP)	CERN OHL v2-S	Available in file repository
couple_reservoir_top	CAD Part (STEP)	CERN OHL v2-S	Available in file repository
couple_reservoir_bottom	CAD Part (STEP)	CERN OHL v2-S	Available in file repository
cap_reservoir_top	CAD Part (STEP)	CERN OHL v2-S	Available in file repository
cap_reservoir_bottom	CAD Part (STEP)	CERN OHL v2-S	Available in file repository
base_sensor	CAD Part (STEP)	CERN OHL v2-S	Available in file repository
sensor_water_level	CAD Part (STEP)	CERN OHL v2-S	Available in file repository
connector_sp13	CAD Assembly (STEP)	CERN OHL v2-S	Available in file repository

Description:

1. shell_reservoir: Nutrient solution container constructed from an 8 PVC SCH40 pipe section (300 mm).
2. couple_reservoir_top: 8 PVC coupler that connects the Reservoir module to the upper stack.
3. couple_reservoir_bottom: 8 PVC coupler used for structural connection at the base of the reservoir.
4. cap_reservoir_top: 8 PVC cap machined used for sealing the internal nutrient tank.
5. cap_reservoir_bottom: 8 PVC cap machined as exterior structural housing for the Reservoir module.
6. base_sensor: Mounting PVC base designed to hold the water-level sensor in place.
7. sensor_water_level: Electronic sensor B0F3WB7X3J used to monitor water level.
8. connector_sp13: SP13 connector utilized for interfacing with the level sensor data.

### 3.6 Growth module

**Fig. 6:**
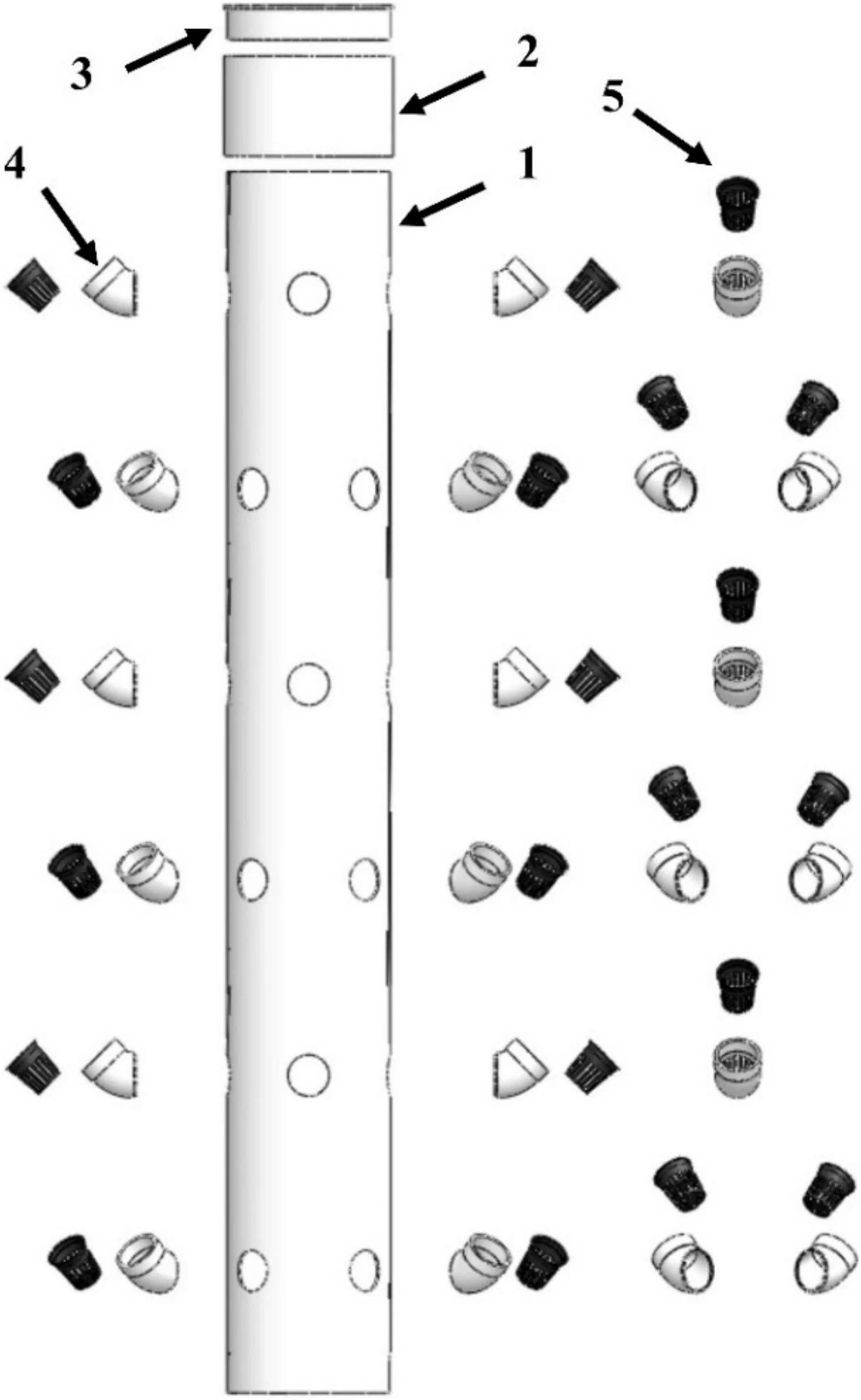
Growth module components: pipe_growth (1), couple_growth (2), cap_growth (3), elbow_020_45 (4), and cup_plant (5).

The constituent parts of the Growth module are listed in Table 7 and illustrated in Fig. 6.

**Table 7:** Design files for the Growth module.

Design filename	File type	Open-source license	Location of the file
pipe_growth	CAD Part (STEP)	CERN OHL v2-S	Available in file repository
couple_growth	CAD Part (STEP)	CERN OHL v2-S	Available in file repository
cap_growth	CAD Part (STEP)	CERN OHL v2-S	Available in file repository
elbow_020_45	CAD Part (STEP)	CERN OHL v2-S	Available in file repository
cup_plant	CAD Part (STEP)	CERN OHL v2-S	Available in file repository

Description:

1. pipe_growth: 1500 mm vertical tower body for cultivation constructed from an 8 PVC SCH40 pipe.
2. couple_growth: Top 8 PVC coupler used to complete the tower structure.
3. cap_growth: Standard 8 PVC cap used to seal the top of the growth tower.
4. elbow_020_45: 45° PVC elbow fitting machined and inserted into the tower to hold plant units.
5. cup_plant: Individual cup that holds the support medium and plant.

### 3.7 Filter module

**Fig. 7:**
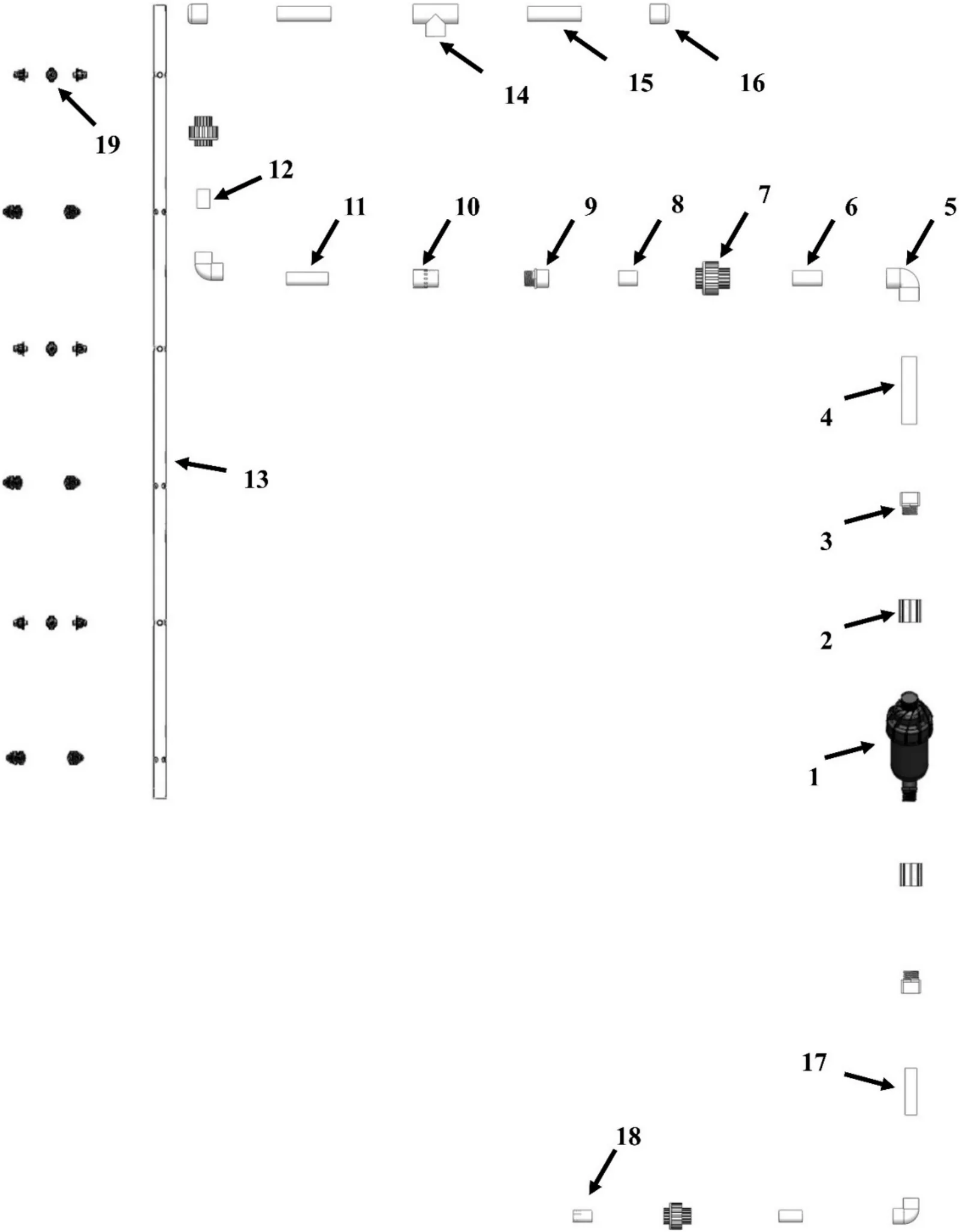
Filter module components: filter_100 (1), coupling_007 (2), adapter_101 (3), pipe_005_94 (4), elbow_005_90 (5), pipe_005_41 (6), union_005 (7), pipe_005_26 (8), adapter_005 (9), coupling_005 (10), pipe_005_59 (11), pipe_005_30 (12), pipe_005_1500 (13), tee_005 (14), pipe_005_75 (15), cap_005 (16), pipe_005_83 (17), pipe_005_33 (18), and nozzle (19).

The constituent parts of the Filter module are listed in Table 8 and illustrated in Fig. 7.

**Table 8:** Design files for the Filter module.

Design filename	File type	Open-source license	Location of the file
filter_100	CAD Assembly (STEP)	CERN OHL v2-S	Available in file repository
coupling_007	CAD Part (STEP)	CERN OHL v2-S	Available in file repository
adapter_101	CAD Part (STEP)	CERN OHL v2-S	Available in file repository
pipe_005_94	CAD Part (STEP)	CERN OHL v2-S	Available in file repository
elbow_005_90	CAD Part (STEP)	CERN OHL v2-S	Available in file repository
pipe_005_41	CAD Part (STEP)	CERN OHL v2-S	Available in file repository
union_005	CAD Assembly (STEP)	CERN OHL v2-S	Available in file repository
pipe_005_26	CAD Part (STEP)	CERN OHL v2-S	Available in file repository
adapter_005	CAD Part (STEP)	CERN OHL v2-S	Available in file repository
coupling_005	CAD Part (STEP)	CERN OHL v2-S	Available in file repository
pipe_005_59	CAD Part (STEP)	CERN OHL v2-S	Available in file repository
pipe_005_30	CAD Part (STEP)	CERN OHL v2-S	Available in file repository
pipe_005_1500	CAD Part (STEP)	CERN OHL v2-S	Available in file repository
tee_005	CAD Part (STEP)	CERN OHL v2-S	Available in file repository
pipe_005_75	CAD Part (STEP)	CERN OHL v2-S	Available in file repository
cap_005	CAD Part (STEP)	CERN OHL v2-S	Available in file repository
pipe_005_83	CAD Part (STEP)	CERN OHL v2-S	Available in file repository
pipe_005_33	CAD Part (STEP)	CERN OHL v2-S	Available in file repository
nozzle	CAD Part (STEP)	CERN OHL v2-S	Available in file repository

Description:

1. filter_100: Filter AZUD MODULAR 100 that removes particles in the water before reaching the nozzles.
2. coupling_007: 3/4 FTP × FTP coupling used to connect the filter core to the transition adapters.
3. adapter_101: 3/4 × 1/2 male MPT × S adapter that transitions the pipeline from the filter.
4. pipe_005_94: 1/2 PVC SCH40 pipe section (94 mm) used in the initial segment of the upward filtration path.
5. elbow_005_90: 1/2 90° S × S elbow used to change flow direction.
6. pipe_005_41: 1/2 PVC SCH40 pipe section (41 mm) used to connect elbows and unions.
7. union_005: 1/2 S × S union that provides a detachable connection point within the central pipeline.
8. pipe_005_26: 1/2 PVC SCH40 pipe segment (26 mm) utilized with the adapters.
9. adapter_005: 1/2 male S × MPT and female S × FPT adapter used for threaded connections.
10. coupling_005: 1/2 S × FTP coupling used for threaded connections.
11. pipe_005_59: 1/2 PVC SCH40 pipe segment (59 mm) located between the adapters and the upper elbow.
12. pipe_005_30: 1/2 PVC SCH40 pipe section (30 mm) that connects the elbow to the upper union.
13. pipe_005_1500: 1500 mm vertical 1/2 PVC SCH40 pipe supporting the misting nozzles.
14. tee_005: 1/2 S × S tee positioned at the top of the misting line to distribute flow to the capped ends.
15. pipe_005_75: 1/2 PVC SCH40 pipe section (75 mm) that extends from the top tee to the sealing cap.
16. cap_005: 1/2 cap used to seal the ends of the distribution line at the top of the tower.
17. pipe_005_83: 1/2 PVC SCH40 pipe segment (83 mm) used for the drainage interface.
18. pipe_005_33: 1/2 PVC SCH40 pipe section (33 mm), machined, glued in the final segment before the drainage exit.
19. nozzle: Aeroponic units (EXL Fogger 89–11) installed along the vertical pipe to provide a fine nutrient mist.

### 3.8 Inlet module

**Fig. 8:**
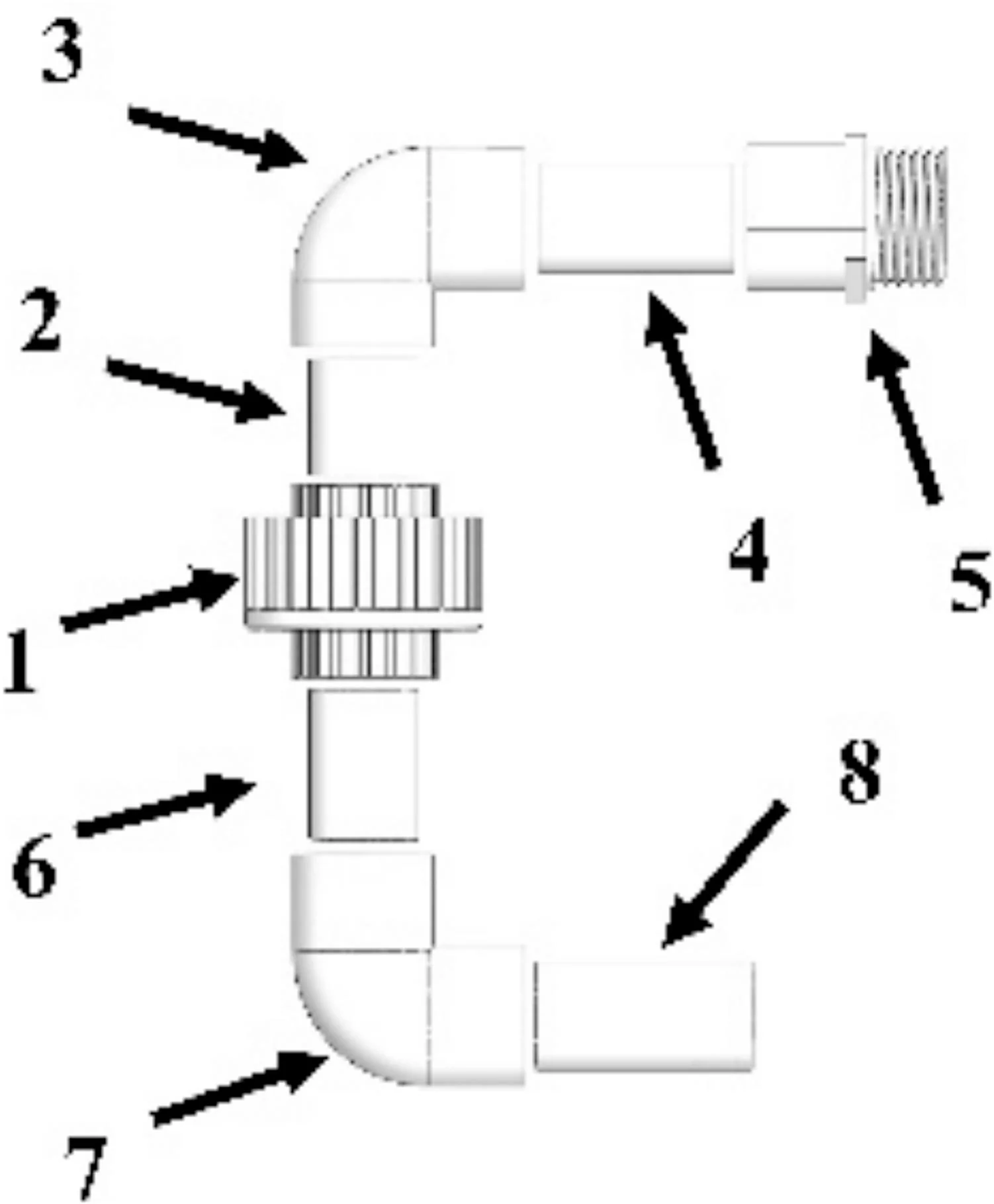
Inlet module components: union_005C (1), pipe_005_22 (2), elbow_005C_90 (3), pipe_005_38 (4), adapter_101 (5), pipe_005_29 (6), elbow_005_90 (7), and pipe_005_42 (8).

The constituent parts of the Inlet module are listed in Table 9 and illustrated in Fig. 8.

**Table 9:** Design files for the Inlet module.

Design filename	File type	Open-source license	Location of the file
union_005C	CAD Part (STEP)	CERN OHL v2-S	Available in file repository
pipe_005_22	CAD Part (STEP)	CERN OHL v2-S	Available in file repository
elbow_005C_90	CAD Part (STEP)	CERN OHL v2-S	Available in file repository
pipe_005_38	CAD Part (STEP)	CERN OHL v2-S	Available in file repository
adapter_101	CAD Part (STEP)	CERN OHL v2-S	Available in file repository
pipe_005_29	CAD Part (STEP)	CERN OHL v2-S	Available in file repository
elbow_005_90	CAD Part (STEP)	CERN OHL v2-S	Available in file repository
pipe_005_42	CAD Part (STEP)	CERN OHL v2-S	Available in file repository

Description:

1. union_005C: 1/2 S × S union machined for detachable connection point within the central pipeline.
2. pipe_005_22: 1/2 PVC SCH40 pipe segment (22 mm) used for the drainage interface.
3. elbow_005C_90: 1/2-inch 90° S × S elbow used to change flow direction.
4. pipe_005_38: 1/2 PVC SCH40 pipe segment (38 mm) used for the drainage interface.
5. adapter_101: 3/4 × 1/2 male MPT × S adapter that transitions the pipeline from the reservoir.
6. pipe_005_29: 1/2 PVC SCH40 pipe segment (29 mm) used for the drainage interface.
7. elbow_005_90: 1/2-inch 90° S × S elbow used to change flow direction.
8. pipe_005_42: 1/2 PVC SCH40 pipe segment (42 mm), machined, used for the drainage interface.

### 3.9 Solar module

**Fig. 9:**
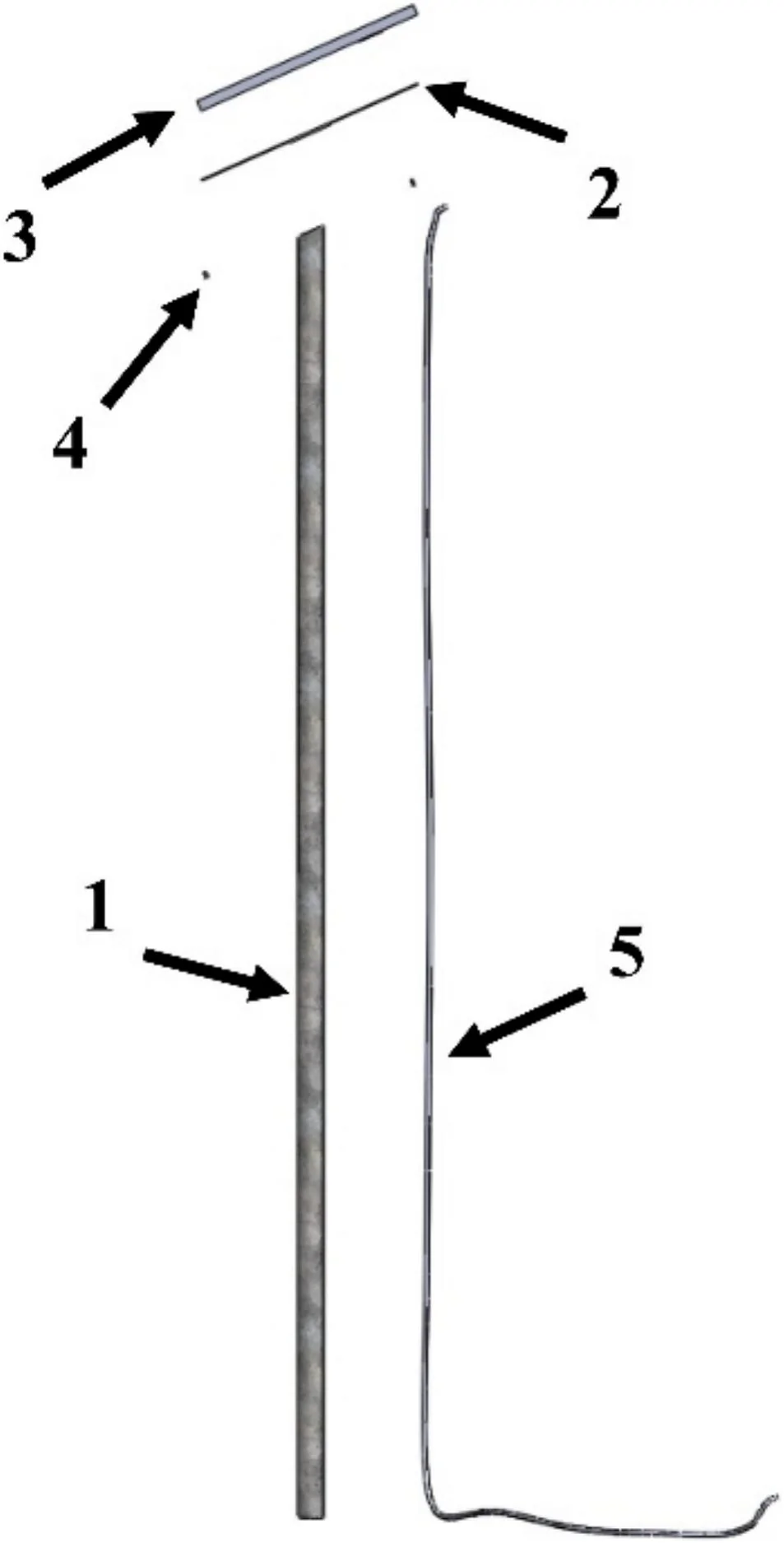
Solar module components: pipe_solar (1), base_panel (2), panel_solar (3), screw_6x10 (4), and cable_solar (5).

The constituent parts of the Solar module are listed in Table 10 and illustrated in Fig. 9.

**Table 10:** Design files for the Solar module.

Design filename	File type	Open-source license	Location of the file
pipe_solar	CAD Part (STEP)	CERN OHL v2-S	Available in file repository
base_panel	CAD Part (STEP)	CERN OHL v2-S	Available in file repository
panel_solar	CAD Part (STEP)	CERN OHL v2-S	Available in file repository
screw_6x10	CAD Part (STEP)	CERN OHL v2-S	Available in file repository
cable_solar	CAD Part (STEP)	CERN OHL v2-S	Available in file repository

Description:

1. pipe_solar: Galvanized pipe (3000 mm) serving as the structural mast for the solar assembly.
2. base_panel: Support structure designed to hold the solar panel, constructed from 1 × 1/8 steel.
3. panel_solar: Polycrystalline solar panel 50 W (PS-50o KF50P-12) used for off-grid power generation.
4. screw_6x10: Screws 6 mm × 10 mm (M6 × 12).
5. cable_solar: 18 AWG 2-conductor 600 V power cable used to supply power from the solar source.

### 3.10 Firmware, electronics, and GUI

ESP32 microcontroller firmware, schematics, and graphical user interface (GUI) codes are listed in Table 11.

**Table 11:** Source files for the TOTEM.

Design filename	File type	Open-source license	Location of the file
TOTEM_ESP32	C++	GNU GPL v3	Available in file repository
TOTEM_SCH	PDF	CERN OHL v2-S	Available in file repository
TOTEM_PCB	PDF	CERN OHL v2-S	Available in file repository
TOTEM_GUI	PHP,HTML,JS,CSS	GNU GPL v3	Available in file repository

Description:

1. TOTEM_ESP32: Firmware for the ESP32 WOOM microcontroller.
2. TOTEM_SCH: Electronic schematic of the printed circuit board for the Control module.
3. TOTEM_PCB: Printed circuit board for the Control module.
4. TOTEM_GUI: Graphical user interface (GUI) code.

## 4 Bill of materials summary

The bill of materials summary reports retail prices obtained in June 2026 for procurement in the United States. The total estimated material cost of USD 719.51 excludes sales tax and shipping charges. Labor, reusable fabrication tools, greenhouse infrastructure, seeds, nutrient solution, internet service, and routine maintenance are also excluded. The reported amount represents a documented material cost estimate for constructing the prototype rather than a fixed acquisition cost. Actual expenditure may vary with supplier, location, purchase date, taxes, and shipping conditions. (See Table 12.).

**Table 12:** Bill of materials for the TOTEM system.

Designator	Component	Number	Unit cost (USD)	Total cost (USD)	Source of materials
base_control	Base plate	1	1.40	1.40	https://8p2cpin.s.gy/TtmrgP
bar_battery	Locking bar	1	1.45	1.45	https://8p2cpin.s.gy/LC6YMC
base_panel	Mounting plate	1	5.84	5.84	https://8p2cpin.s.gy/781uUf
battery_12V	12 V 12Ah rechargeable battery	2	28.49	56.98	https://8p2cpin.s.gy/jykYVz
adapter_LFA385	1/2 NPT brass adapter	2	6.57	13.14	https://8p2cpin.s.gy/vzUC7r
cable_pump	22 AWG 2-wire cable	1	0.28	0.28	https://8p2cpin.s.gy/ywRu4h
cable_level	22 AWG 2-wire cable	1	0.47	0.47	https://8p2cpin.s.gy/ywRu4h
cable_solar	18 AWG 2-wire cable	1	19.00	19.00	https://8p2cpin.s.gy/4P3Tlv
sensor_temperature	DS18B20 waterproof sensor	1	9.95	9.95	https://8p2cpin.s.gy/BVDmZN
terminal	6 × 2 terminal block	1	6.49	6.49	https://8p2cpin.s.gy/7VEMGc
connector_sp13	SP13 waterproof connector	9	3.29	29.61	https://8p2cpin.s.gy/tTgYrC
optocoupler_FOD852	FOD852 optocoupler	1	1.34	1.34	https://8p2cpin.s.gy/5HiT7i
relay_RAS1220M	12 V relay switch	1	0.75	0.75	https://8p2cpin.s.gy/lMHlJf
controller	40A solar charge controller	1	21.99	21.99	https://8p2cpin.s.gy/Ti8Cam
hose	1/2 irrigation hose	2	0.20	0.40	https://8p2cpin.s.gy/PRgrl1
pcb_control	Custom printed circuit board	1	1.99	1.99	https://8p2cpin.s.gy/E44NVi
esp32_wroom	ESP32 WROOM microcontroller	1	6.33	6.33	https://8p2cpin.s.gy/nEhqPT
filter_100	Filtration mesh	1	46.06	46.06	https://8p2cpin.s.gy/uigWkT
cup_plant	Net cups for plants	24	1.49	35.76	https://8p2cpin.s.gy/Nxpger
nozzle	Aeroponic mist nozzle	24	0.97	23.28	https://8p2cpin.s.gy/OoMD0i
regulator_5V	LM2596 voltage regulator	1	1.29	1.29	https://8p2cpin.s.gy/lb41ux
water_pump	12 V DC water pump	1	32.99	32.99	https://8p2cpin.s.gy/Hhbf1I
panel_solar	Photovoltaic panel 50 W	1	79.00	79.00	https://8p2cpin.s.gy/dvXc6l
base_sensor	Sensor mounting base	1	1.32	1.32	https://8p2cpin.s.gy/FoSSyJ
couples	8 PVC coupler control housing	5	2.89	14.45	https://8p2cpin.s.gy/rBAqgc
caps	8 PVC end cap	6	7.66	45.96	https://8p2cpin.s.gy/ZDL4Qg
pipe_080	8 PVC pipe section (2770 mm)	1	44.58	44.58	https://8p2cpin.s.gy/QdgnYC
pipe_005	1/2 PVC pipe (2199 mm)	1	4.03	4.03	https://8p2cpin.s.gy/fu7SfI
union_005	1/2 union connector	3	8.65	25.95	https://8p2cpin.s.gy/kuk0y9
elbow_020	45° plant holder elbow	24	4.00	96.00	https://8p2cpin.s.gy/qqRhdQ
elbow_005	1/2 90° elbow	5	2.49	12.45	https://8p2cpin.s.gy/dOc1SE
coupling_005	1/2 threaded coupling	1	0.96	0.96	https://8p2cpin.s.gy/dOc1SE
coupling_007	3/4 FTP coupling	2	1.67	3.34	https://8p2cpin.s.gy/GmPNDW
adapter_101	3/4 × 1/2 MPT adapter	3	1.17	3.51	https://8p2cpin.s.gy/7XLuoi
adapter_005	1/2 threaded adapter	1	0.65	0.65	https://8p2cpin.s.gy/kk3uwT
tee_005	1/2 PVC tee	1	0.99	0.99	https://8p2cpin.s.gy/wVQUvM
cap_005	1/2 PVC cap	2	0.30	0.60	https://8p2cpin.s.gy/SmdpZs
sensor_water_level	Water level sensor	1	11.77	11.77	https://8p2cpin.s.gy/OPZX4X
pipe_solar	Solar mast pipe (3000 mm)	1	36.76	36.76	https://8p2cpin.s.gy/dOgTPL
nut_M6	M6 hex nuts	2	0.41	1.64	https://8p2cpin.s.gy/4gL6zU
wingnut_M6	M6 wing nuts	2	6.22	12.44	https://8p2cpin.s.gy/HhWrf8
screw_3x6	M3 × 6 mm screws	11	0.12	1.32	https://8p2cpin.s.gy/8UO1BB
screw_3x12	M3 × 12 mm screws	4	0.12	0.48	https://8p2cpin.s.gy/SUNx2H
screw_6x10	M6 × 10 mm screws	4	0.48	1.90	https://8p2cpin.s.gy/n5jtlY
pole_battery	1/4 steel threaded rod	2	2.92	5.84	https://8p2cpin.s.gy/VwGG8v
		Total		719.51	

## 5 Build instructions

**Fig. 10:**
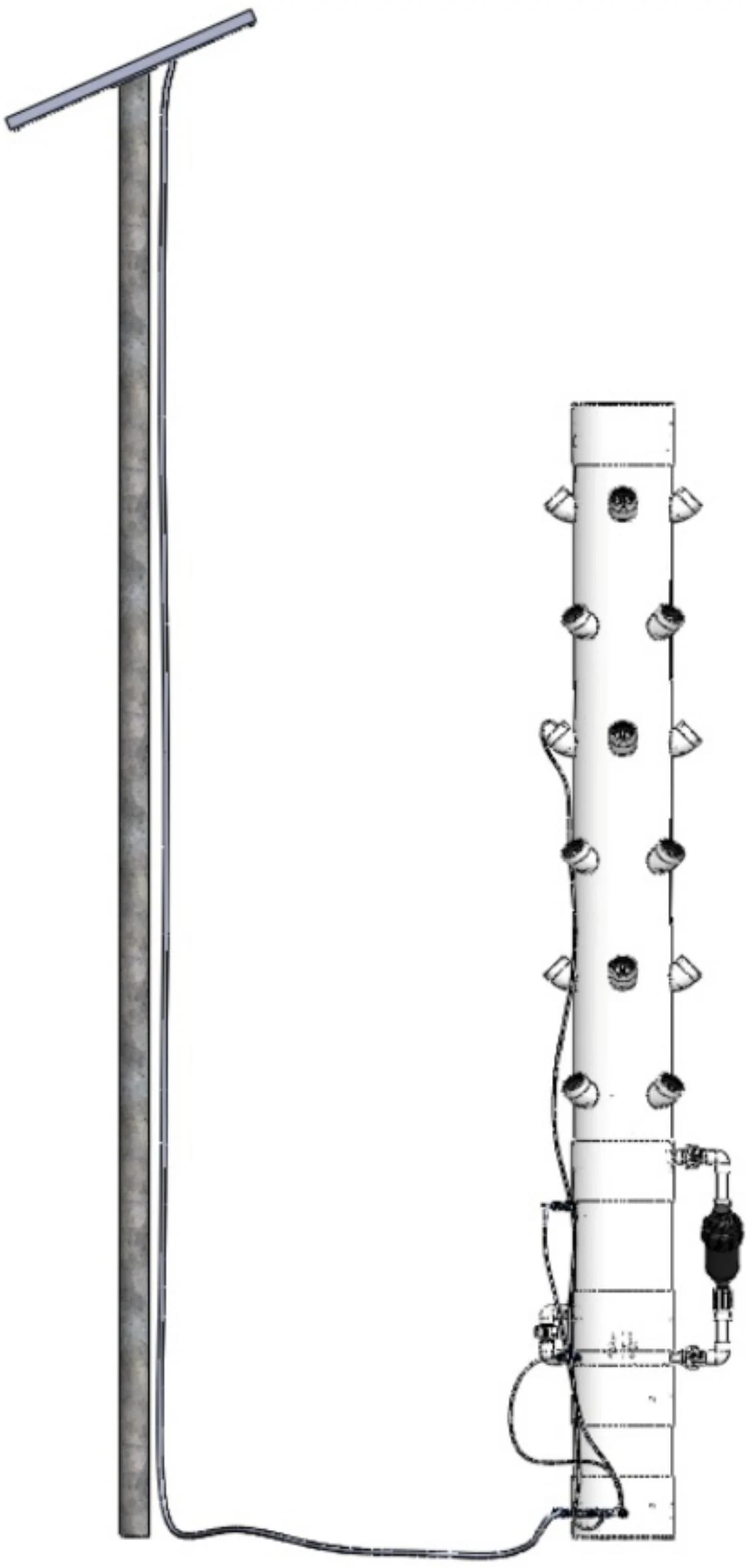
Low-cost solar aeroponic TOTEM system.

The TOTEM system is designed as a vertically stacked architecture composed of discrete functional modules (Fig. 10). Each module is fabricated using standard, accessible materials, such as PVC pipes, couplers, and caps of uniform diameter, enabling reproducibility, replacement, and adaptation across diverse settings. Modules are integrated into a system using mechanical fittings and electrical connections. Assembly is performed in two stages: (i) module fabrication and (ii) system integration. Unless otherwise stated, a diameter of 0.20 m (8 in) is used for all housing modules described below, whereas a diameter of 0.0127 m (1/2 in) is used for misting, filtration, and drainage elements.

### 5.1 Control module

Construction begins with preparation of the housing by sealing a cap (cap_control) to the base of a coupler (couple_control) using PVC solvent cement. Openings are drilled to accommodate waterproof SP13 female connectors.

**Fig. 11:**
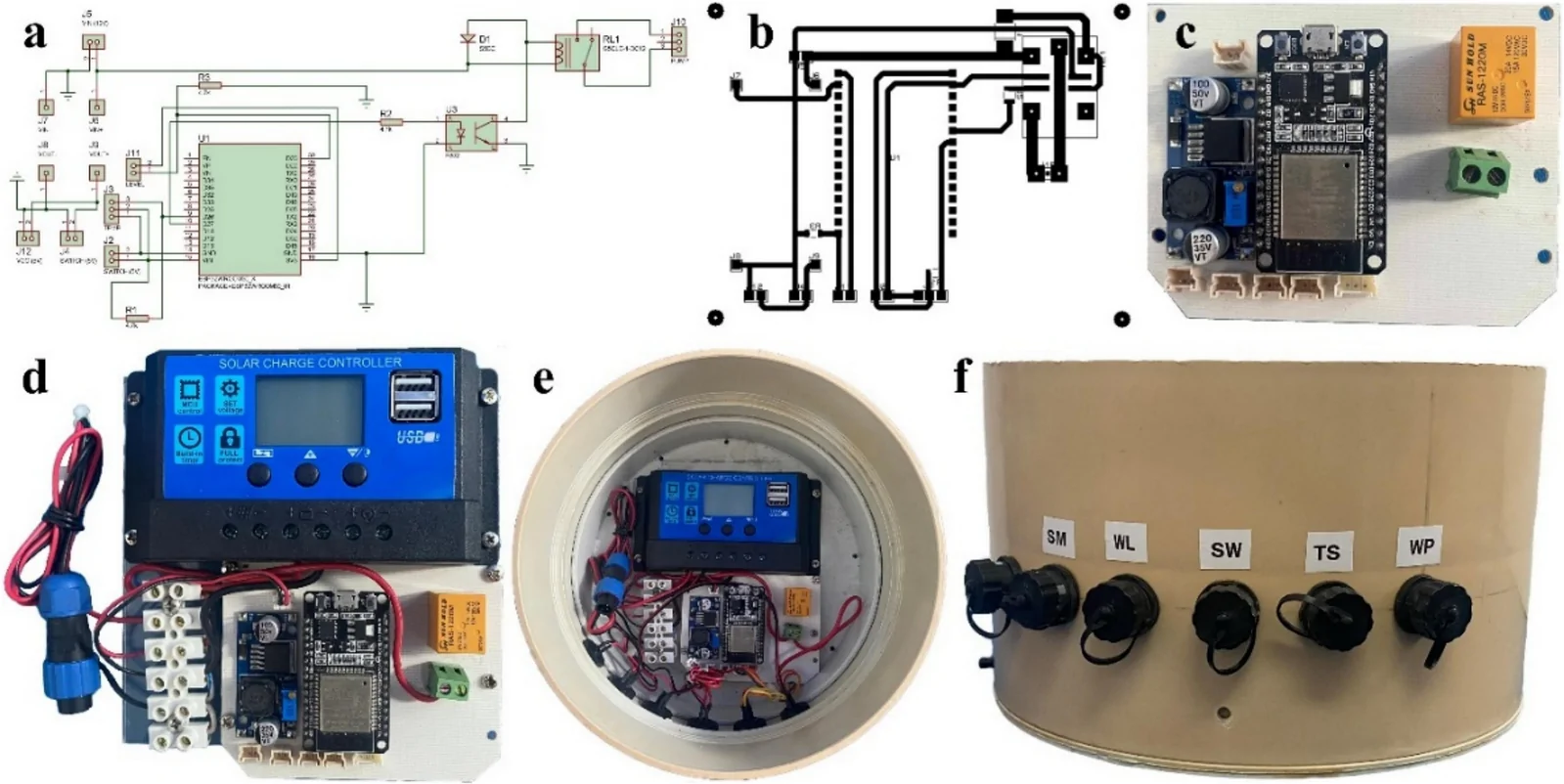
Control module: (a) circuit schematics; (b) PCB design; (c) electronic components; (d) control base assembly; (e) control housing; and (f) completed module, labels indicate interfaces to the Solar module (SM), water-level sensor (WL), in-built switch (SW), temperature sensor (TS), and water pump (WP).

The electronic control core is assembled according to the circuit schematics (Fig. 11a) and implemented on an in-house-designed PCB (Fig. 11b). The PCB integrates an ESP32-WROOM microcontroller, an LM2596 voltage regulator board (5 V output), and an RAS1220M relay for pump actuation via a FOD852 optocoupler. Additional headers and terminals provide interfaces for sensors and external modules (Fig. 11c).

The PCB, a 40 A MPPT charge controller, and a 6 × 2 terminal block are mounted on an acrylic base plate using M3 screws (Fig. 2d), forming a compact power distribution and control unit. The charge controller regulates energy from the photovoltaic panel and supplies power to both the battery and the control electronics.

Electrical connections are established by soldering cables from system inputs and outputs (Solar module, sensors, relay, and regulator) to the corresponding SP13 connectors mounted on the housing (Fig. 11e). The assembled electronics are then inserted into the enclosure, completing the module and providing environmental protection (Fig. 11f).

Before system integration, functionality is verified by checking voltage outputs (12 V and 5 V), sensor data, and relay switching.

### 5.2 Power module

**Fig. 12:**
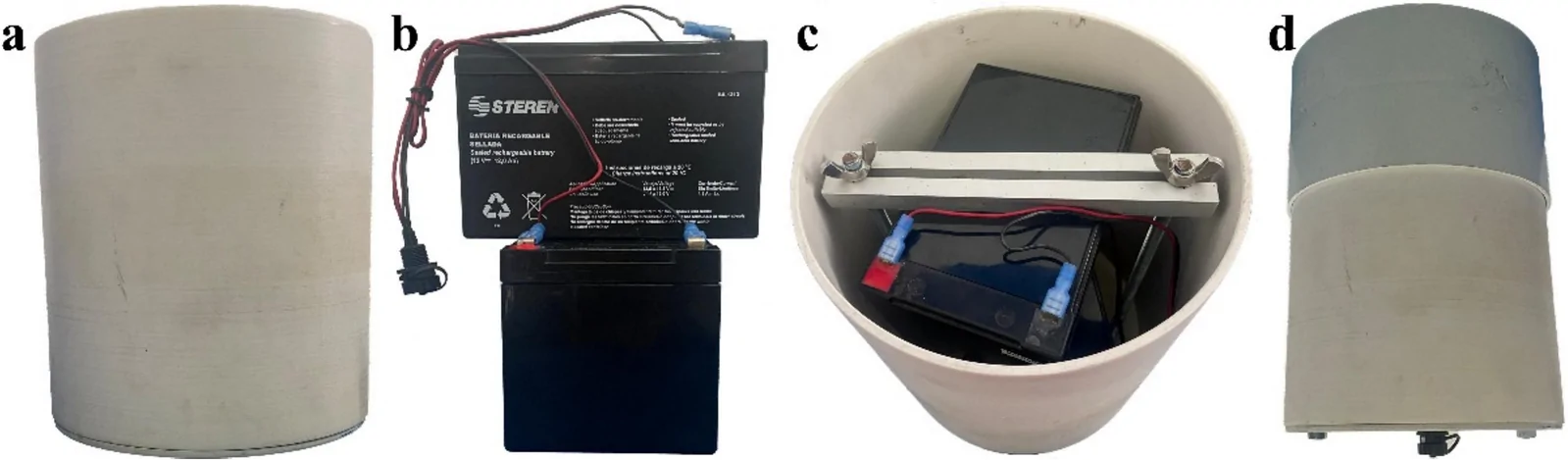
Power module: a) power housing with sealed cap; b) parallel battery pack; c) poles and bar stacked inside and SP13 female connector; and d) assembly Power module.

The battery housing is prepared by removing the insertion section from a cap (cap_power). Openings are drilled to accommodate a waterproof SP13 female connector and two steel rods. The cap is fitted to a pipe section (shell_power, approximately 220 mm long), which provides structural support and ensures the alignment of internal components (Fig. 12a).

Two 12 V–12 Ah rechargeable batteries are configured in parallel using an electrical wiring harness to maintain a 12 V output while increasing storage capacity (Fig. 12b). Electrical connections are soldered to the SP13 connector terminals and to the battery terminals. The connector is installed through the cap opening to enable secure interfacing with the Control module.

The batteries are positioned vertically within the enclosure and guided by two threaded steel rods (pole_battery) that pass through the cap and are secured with hex nuts. An aluminum locking bar (bar_battery) is installed across the top of the battery stack and tightened with wing nuts to stabilize the assembly (Fig. 12c). Final enclosure is achieved by installing an external coupler (couple_power), which completes the protective housing (Fig. 12d).

### 5.3 Pump module

**Fig. 13:**
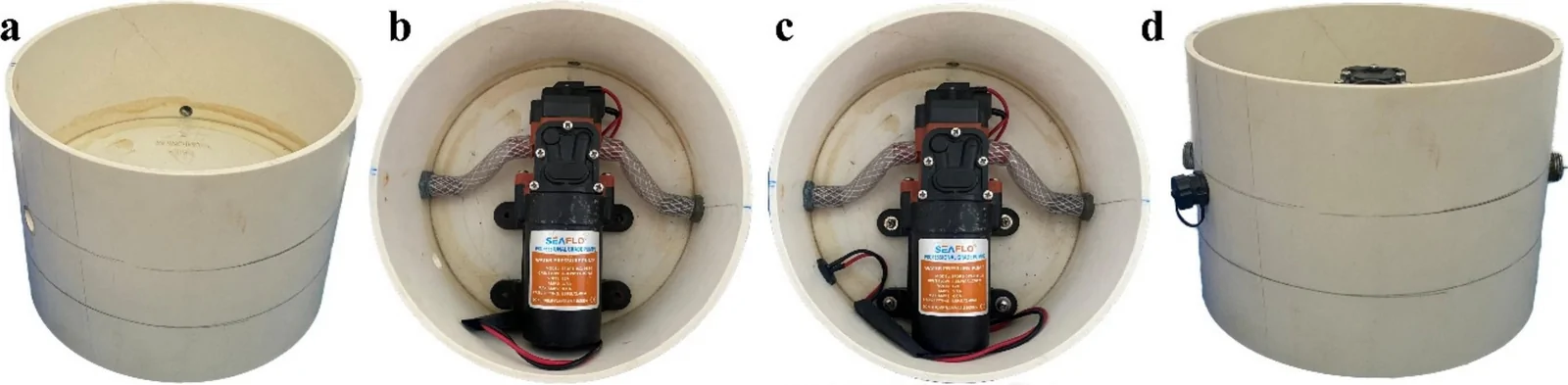
Pump module: a) pump housing; b) inlet and outlet ports; c) female connector; and d) pumping assembly.

The housing is prepared using a pipe section (shell_pump, approximately 150 mm in length), which forms the enclosure for the irrigation pump (Fig. 13a). Openings are drilled to accommodate inlet and outlet adapters and a waterproof SP13 female connector. A machined cap (cap_pump), modified by removing its insertion section, serves as the pump mounting base and is bonded to the housing midsection.

The irrigation pump is positioned inside the enclosure and over the cap. The inlet and outlet ports are connected to the fluid circuit using NPT brass adapters (adapter_LFA385) and flexible tubing, which route the nutrient solution between the Reservoir and Filter modules (Fig. 13b).

Electrical wiring from the pump is routed through the SP13 connector installed in the housing (Fig. 13c). The pump is actuated via a relay circuit controlled by the microcontroller. Following verification of hydraulic and electrical connections, the pump is fixed in place using M3 × 12 screws, completing the module assembly (Fig. 13d).

### 5.4 Reservoir module

**Fig. 14:**
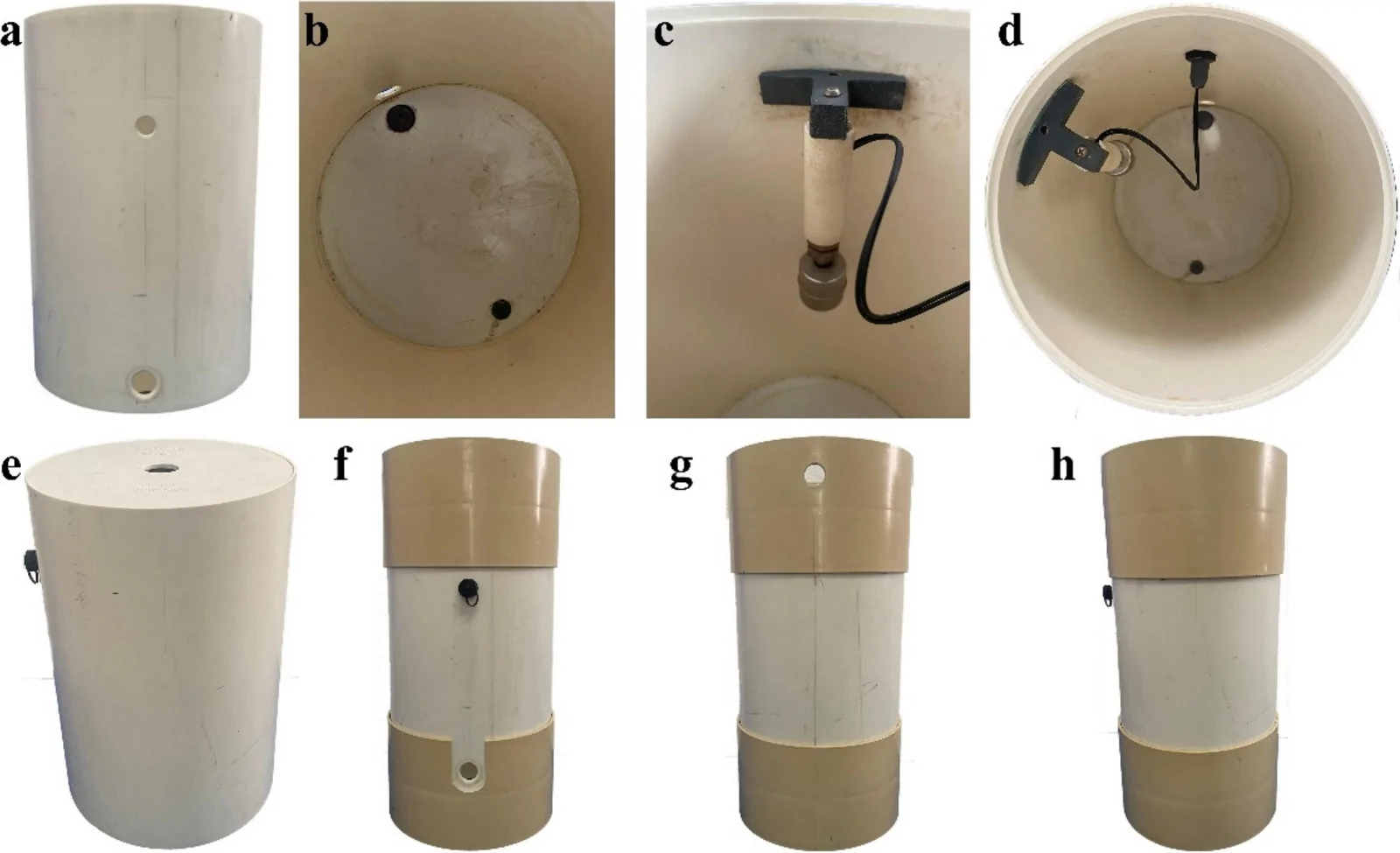
Reservoir module: a) reservoir housing; b) sealed bottom chamber; c) water level sensor; d) female connector; e) closed top chamber; f and g) top and bottom couplers; h) reservoir assembly.

A pipe section (shell_reservoir, approximately 300 mm in length) is used to form the nutrient solution chamber. Openings are drilled to accommodate inlet and outlet drain connections and a waterproof SP13 female connector (Fig. 14a). The lower end is sealed with a machined cap (cap_reservoir_bottom), bonded in place to form the base of the reservoir (Fig. 14b).

The water-level sensing assembly is then installed. A dedicated mounting component (base_sensor) is bonded inside the chamber to support the water-level sensor (Fig. 14c). Sensor wiring is routed to a female connector, enabling interfacing with the Control module for monitoring nutrient solution levels (Fig. 14d).

The chamber is closed by installing a machined top cap (cap_reservoir_top), which includes a central opening to allow return flow from the growth tower (Fig. 14e). The cap is sealed using silicone to permit future maintenance access.

Structural couplers are subsequently prepared and installed. A bottom coupler (couple_reservoir_bottom) is machined with an opening for connection to the Inlet module (Fig. 14f), whereas an upper coupler (couple_reservoir_top) is modified to interface with the Filter module (Fig. 14g). Both couplers are installed to enable integration with adjacent modules in the stacked system (Fig. 14h).

### 5.5 Growth module

A vertical pipe section (pipe_growth, 1.5 m in length) forms the structural body of the cultivation tower. Openings for plant sites are drilled along the circumference, distributed at 90° intervals across six staggered rows, with a 45° offset between rows and a vertical spacing of 0.22 m.

**Fig. 15:**
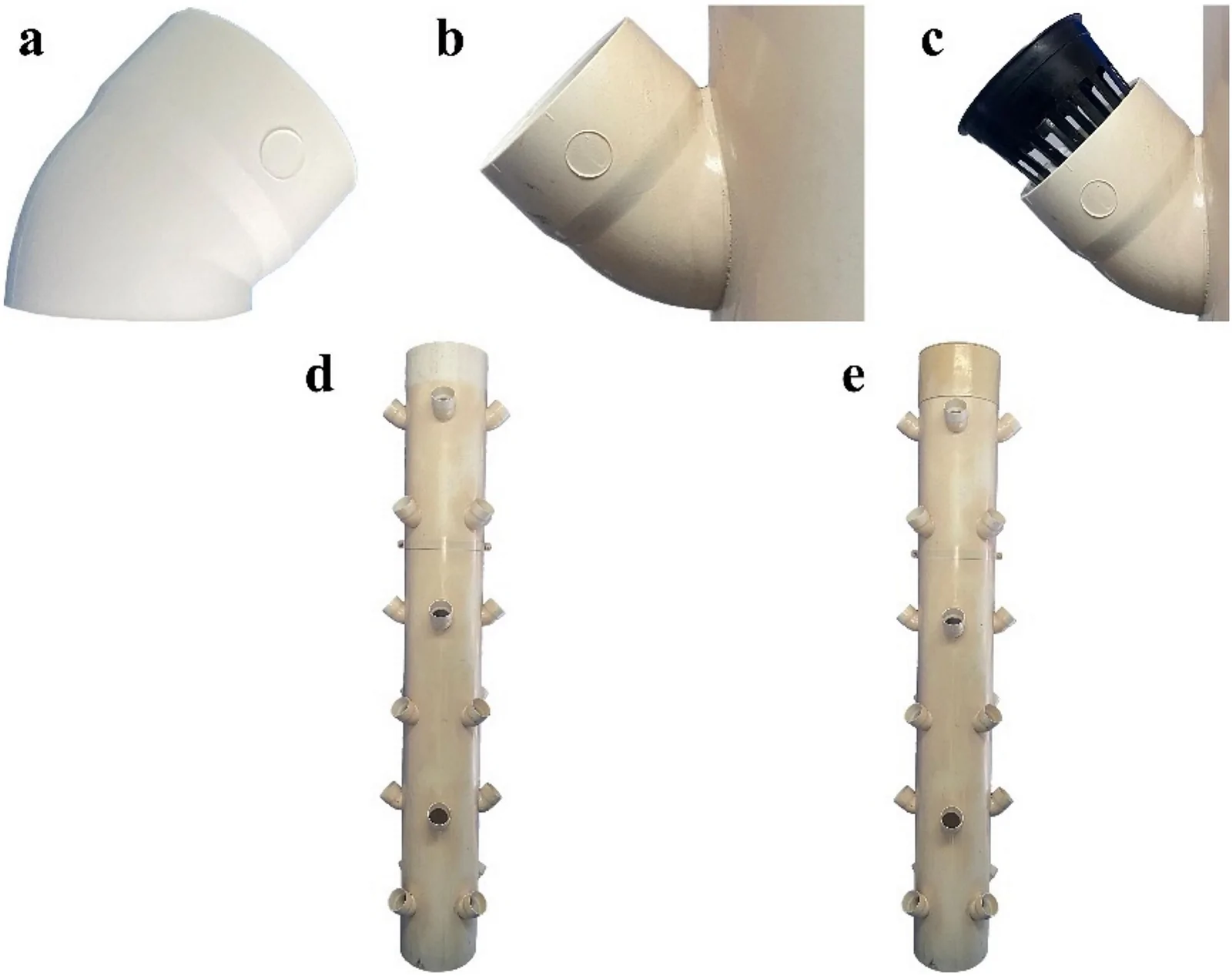
Growth module: a) plant holders; b) plant holders glued; c) plastic net cup; d) 24 plant sites; and e) growth assembly.

A total of 24 angled plant holders are fabricated by machining 45° PVC elbows (elbow_020_45) and removing a portion of each socket section (Fig. 15a). Each modified elbow is inserted into a corresponding opening and secured with adhesive, forming an inclined base that allows plant roots to extend into the interior while maintaining stable positioning of the containers (Fig. 15b).

Each elbow is fitted with a plastic net cup (cup_plant), which holds the plant and its support medium (Fig. 15c). In total, 24 plant sites are distributed along the tower to maximize cultivation density within the compact structure (Fig. 15d).

The top section is completed by attaching a coupler (couple_growth) and sealing it with a cap (cap_growth). These components provide structural stability and enable integration with the internal misting system supplied by the Filter module (Fig. 15e).

### 5.6 Filter module

**Fig. 16:**
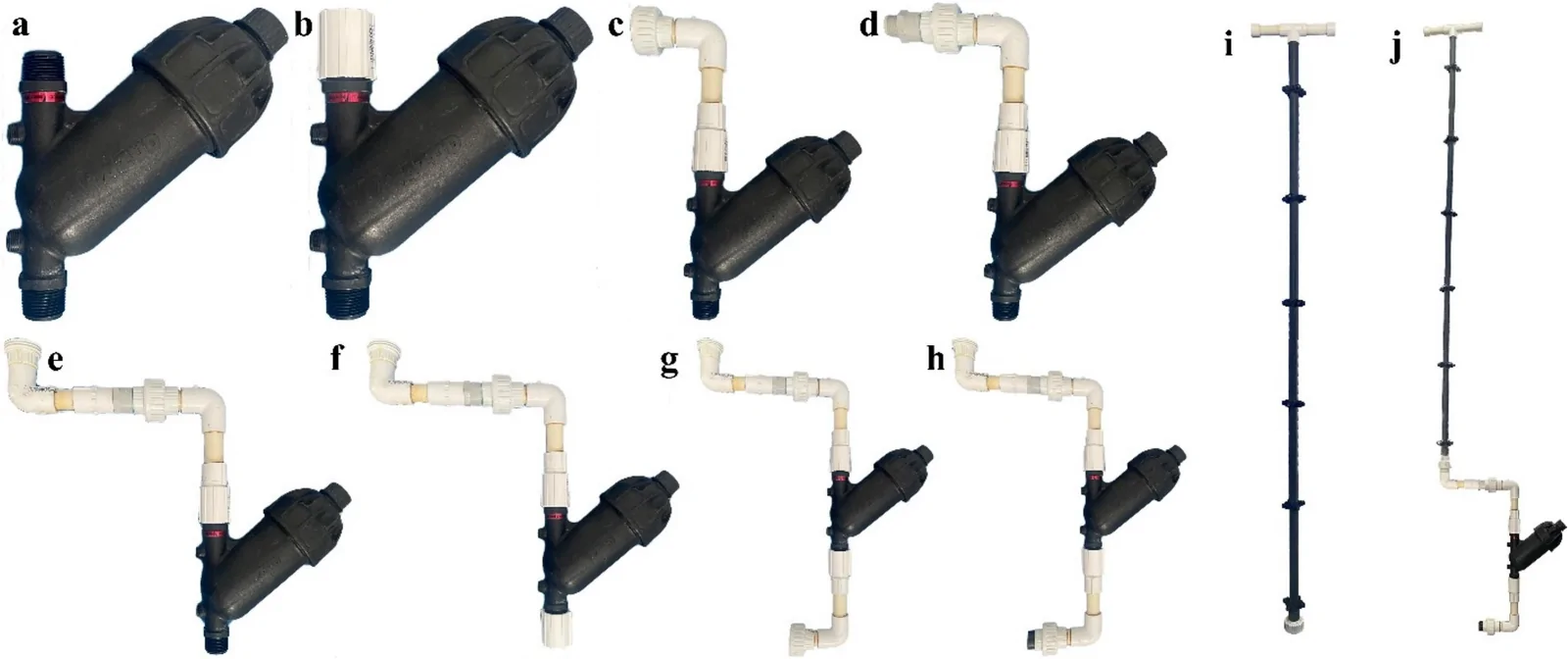
Filter module: a) mesh filter; b) upward coupler; c) horizontal change direction section; d) upward detachable section; e) vertical change direction section; f) downward coupler; g) downward change direction section; h) downward detachable section; i) detachable misting pipeline; and j) filter assembly.

The filtration unit is based on an AZUD MODULAR 100 filter (filter_100), which removes particulates to prevent clogging of the misting nozzles (Fig. 16a). In this module, all PVC piping segments use a diameter of 0.0127 m (1/2 in).

The upward distribution pipeline is assembled using threaded couplings (coupling_007), followed by installation of a 3/4 × 1/2 MPT × S adapter (adapter_101), a pipe segment (94 mm), a 90° elbow (elbow_005_90), a pipe segment (41 mm), and the male nut of a detachable union (union_005) (Fig. 16b–c). The assembly continues with the corresponding female union, a pipe segment (26 mm), a male S × MPT adapter (adapter_005), and a female S × FPT coupler (coupling_005). Additional segments include a pipe section (59 mm), a 90° elbow, a pipe segment (30 mm), and a detachable union to complete the upward path (Fig. 16d–e). The downward path originates at the filter coupling and includes an adapter, a pipe segment (83 mm), a 90° elbow, a pipe segment (41 mm), and a detachable union (Fig. 16f–g). The line is completed with the corresponding union end, and a pipe segment (33 mm) with an internal thread connected to the drainage interface (Fig. 16h). These fittings enable directional flow changes and provide accessible disconnection points for maintenance.

A vertical distribution pipe (pipe_005_1500, 1.5 m in length) extends through the Growth module and supports the aeroponic misting system. A total of 24 misting nozzles are installed along this pipe to deliver nutrient mist to each plant root zone. A detachable union is installed below the distribution line to facilitate modular disassembly. The top section is completed using a tee (tee_005), short pipe segments (75 mm), and sealed caps (cap_005) to center the pipe inside the growth tower (Fig. 16i). Final assembly is achieved by connecting the pipeline to the distribution network (Fig. 7j).

### 5.7 Inlet module

The inlet–drainage assembly connects the Reservoir module to the Pump module through a detachable union connector (union_005C), which serves as the central junction. In this module, all PVC piping components use a diameter of 0.0127 m (1/2 in).

**Fig. 17:**
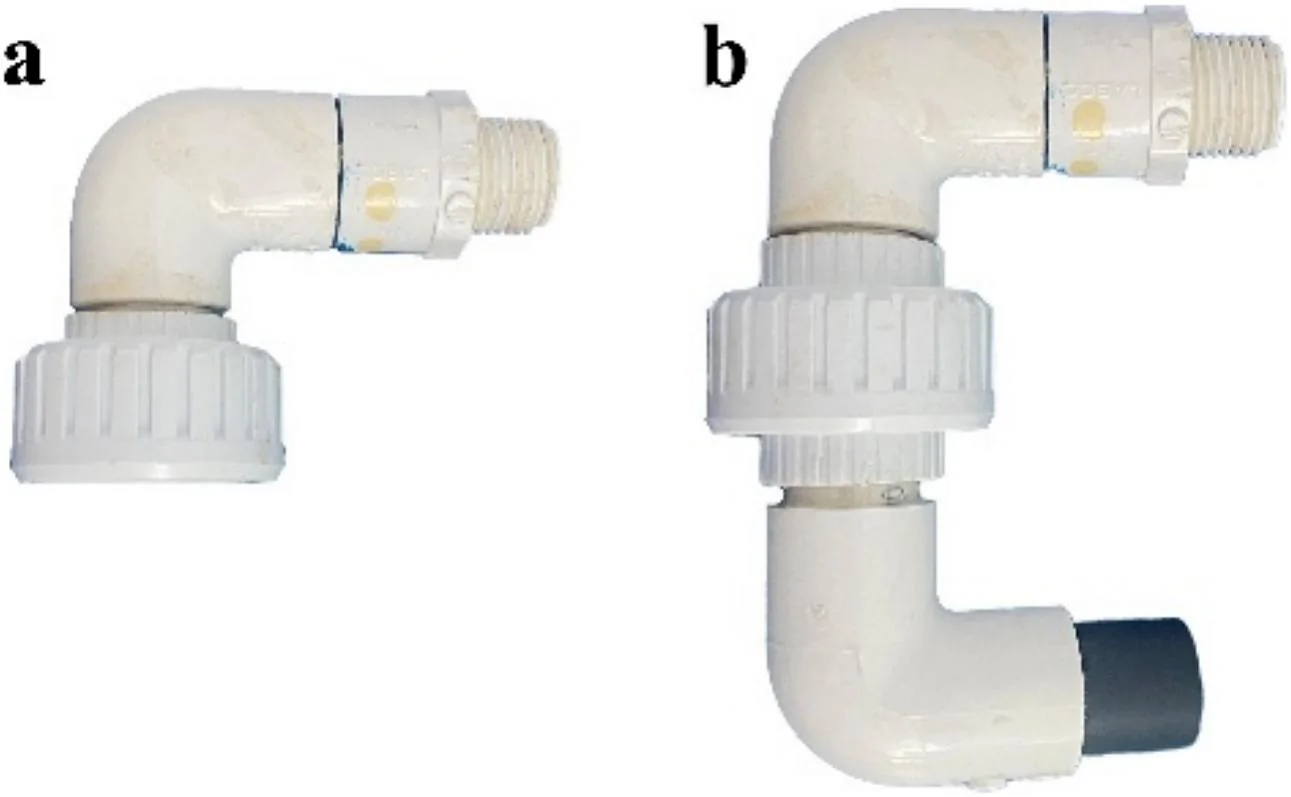
Inlet module: a) upward detachable section; and b) lower detachable section.

The upper intake line is assembled by sequentially connecting a short pipe section (26 mm), a 90° elbow (elbow_005C_90), a second pipe section (26 mm), and a male S × MPT adapter (adapter_101) (Fig. 17a).

The lower drainage path extends from the bottom port of the union. It consists of a pipe section (26 mm), a 90° elbow, and an additional pipe section (26 mm) with an internal thread, completing the inlet–drainage assembly (Fig. 17b).

### 5.8 Solar module

A galvanized steel pipe (pipe_solar, approximately 3000 mm long) serves as the structural mast to elevate the photovoltaic panel and maximize exposure to sunlight.

The mounting frame (base_panel) is fabricated from galvanized steel angle sections to support the panel. The top end of the mast is cut at a 24° angle, allowing the frame to be securely seated and properly oriented for solar incidence. A 50 W polycrystalline photovoltaic panel (panel_solar) is attached to the frame using M6 × 10 fasteners.

Electrical power generated by the panel is transmitted through an 18-AWG two-conductor cable (cable_solar), routed along the mast, and connected to the Control module, where a charge controller regulates battery charging.

**Fig. 18:**
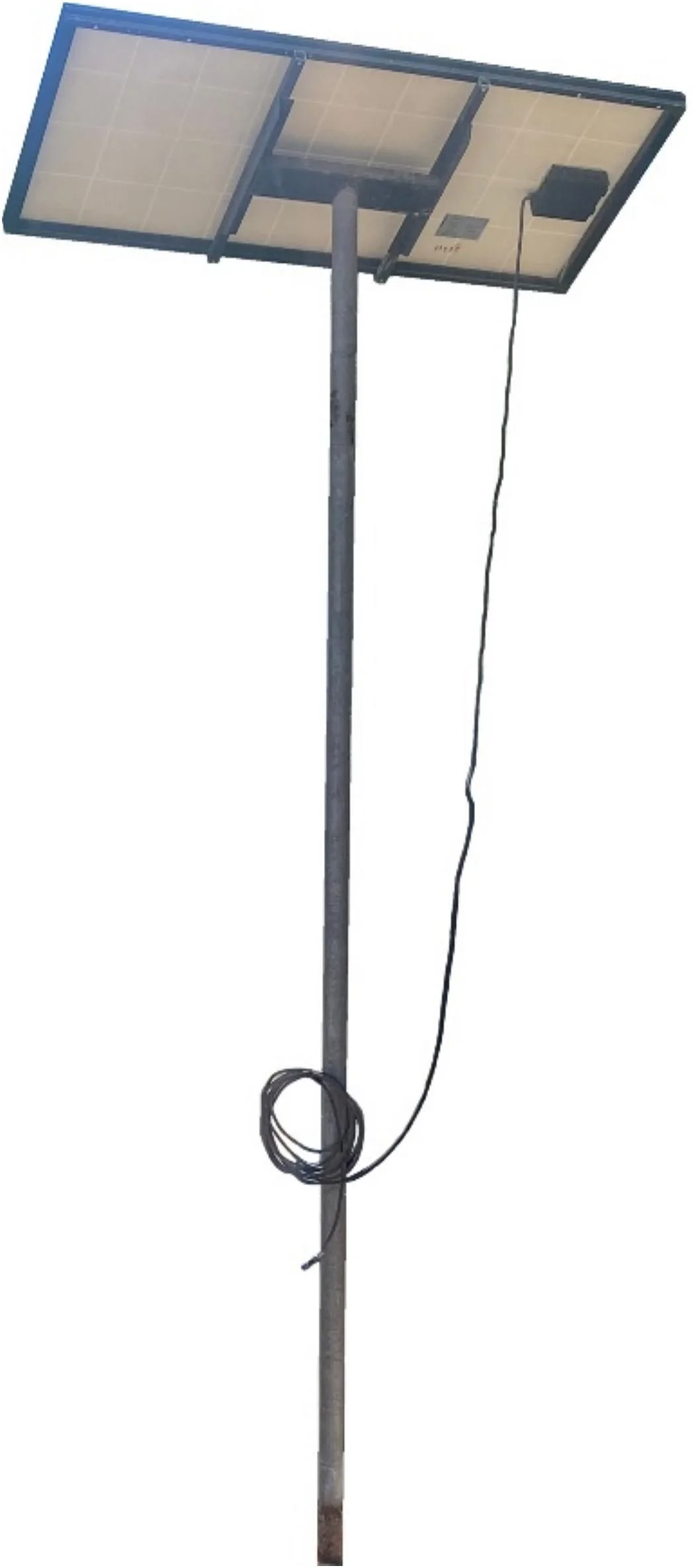
Solar module assembly based on a galvanized steel pipe, a mounting frame, and a solar panel.

The assembled structure is oriented southward to optimize solar exposure, completing the module and enabling renewable energy supply to the system (Fig. 18).

### 5.9 System integration

**Fig. 19:**
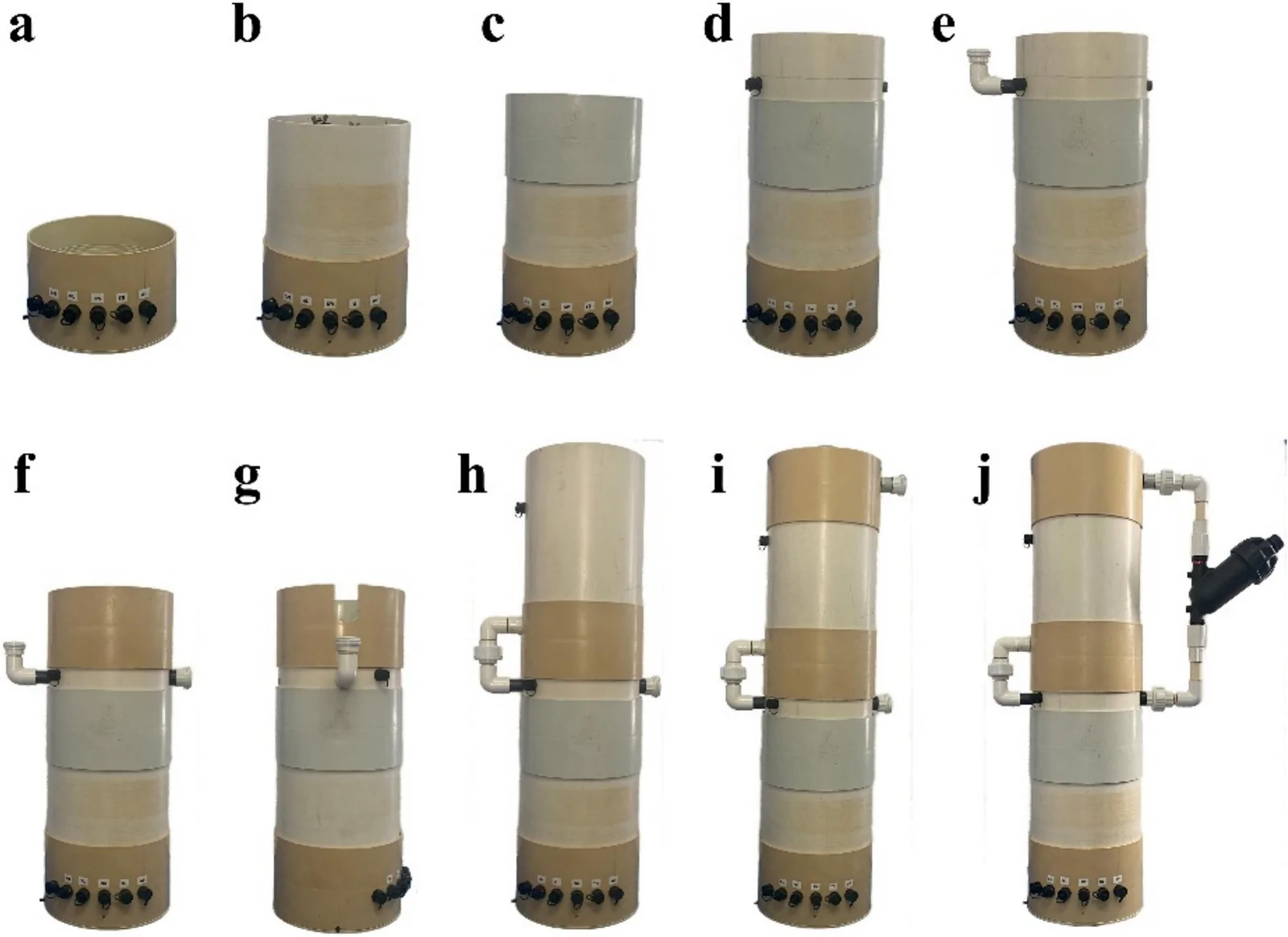
TOTEM: a) Control module; b) power housing; c) Power module; d) pump housing; e) inlet port; f) outlet port; g) lower reservoir coupler (lateral view); h) reservoir housing; i) upper reservoir coupler; and j) filtration assembly.

The TOTEM system is assembled by vertically stacking the modules in a defined sequence. The Control module is positioned as the foundation (Fig. 19a), followed by installation of the Power module (Fig. 19b–c) and the Pump module (Fig. 19d).

The lower drainage line of the Inlet module is then attached (Fig. 19e). The female union from the Filter module is installed next (Fig. 19f), followed by connection to the bottom coupler of the Reservoir module (Fig. 19g). The reservoir is assembled. The Inlet module is secured in place (Fig. 19h).

The top coupler of the Reservoir module is installed together with the upward-facing female union of the Filter module (Fig. 19i). The remaining components of the Filter module are then attached to complete the filtration assembly (Fig. 19j).

**Fig. 20:**
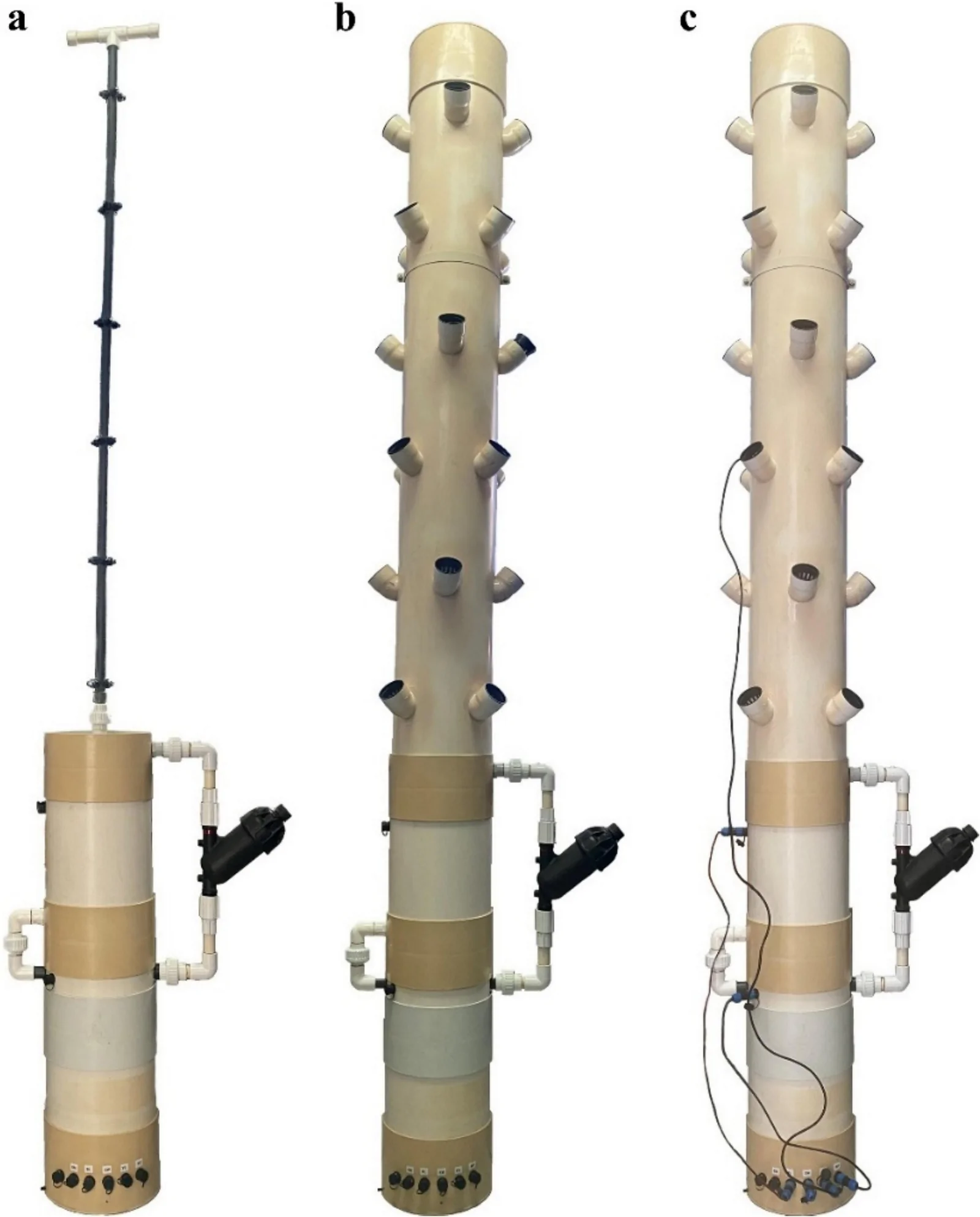
TOTEM: a) vertical misting pipeline; b) Growth module; and c) wiring system.

The vertical misting pipe is assembled and installed (Fig. 20a). Finally, the Growth module is placed at the top of the stack, completing the aeroponic structure (Fig. 20b). Electrical connections are finalized, completing the system assembly (Fig. 20c).

Fabrication was performed using conventional workshop tools selected for each operation. PVC tubes and structural components were cut using a PVC pipe cutter and a power saw, while openings for fittings, connectors, and nozzles were produced with bench-mounted or handheld drills equipped with twist drill bits and hole saws. Files, a deburring tool, and abrasive paper were used to finish the cut edges, and dimensional adjustments were made using a conventional lathe and milling machine. PVC joints were cleaned and joined using a compatible solvent cement. Electronic assemblies were completed using a temperature-controlled soldering iron, solder, and standard PCB hand tools. Low-voltage wiring was performed using wire cutters, wire strippers, crimping pliers, insulated screwdrivers, and a digital multimeter. Final assembly was completed using screwdrivers and other hand tools appropriate for the M3 and M6 fasteners. All fabrication and assembly operations were performed using appropriate personal protective equipment.

## 6 Operation instructions

**Fig. 21:**
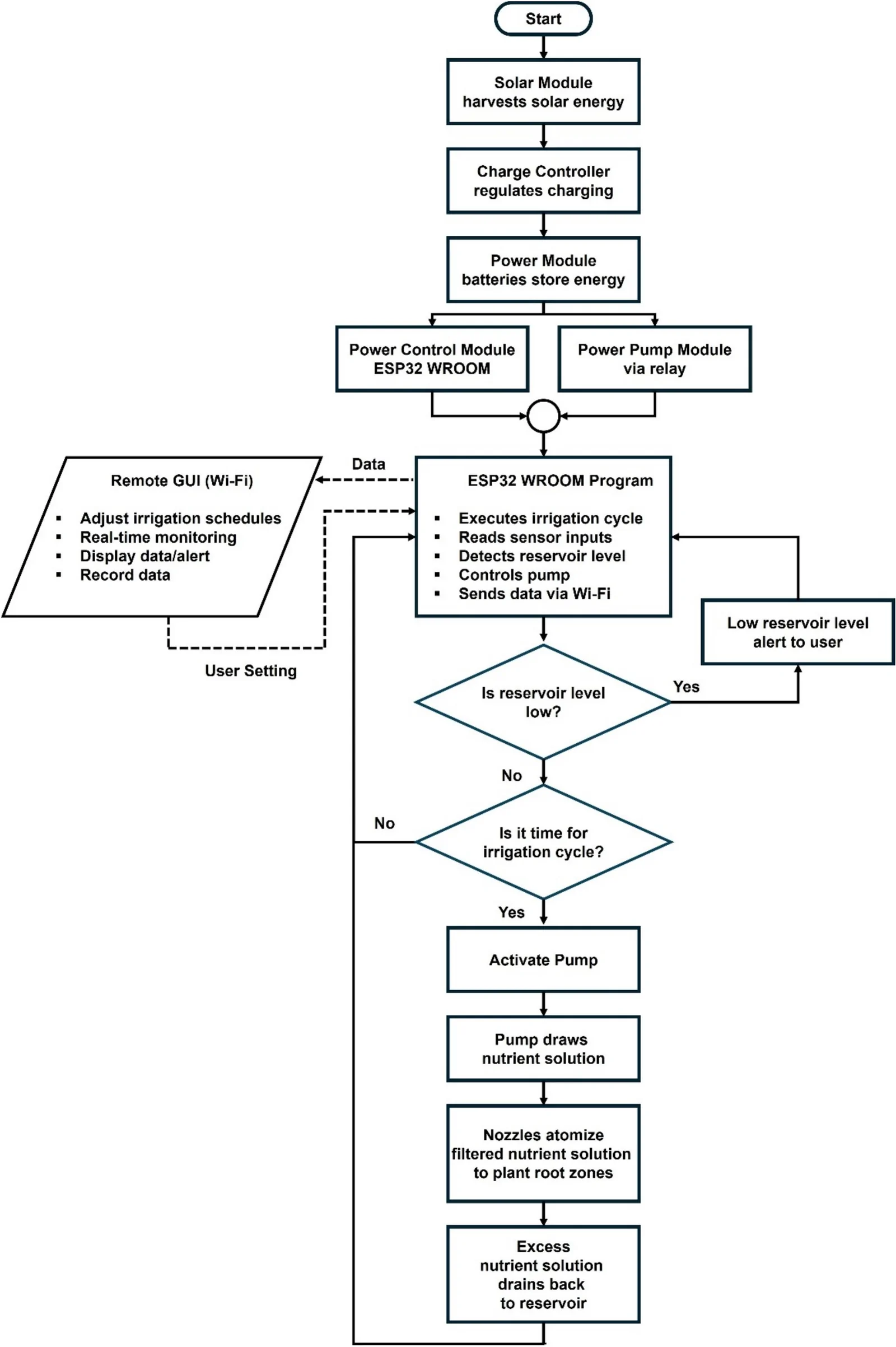
TOTEM operation: logical integration of the aeroponic platform that combines plant cultivation, nutrient delivery, energy autonomy, and automated control.

The TOTEM system operates as a closed-loop, solar-powered aeroponic platform integrating energy management, automated irrigation, and real-time monitoring (Fig. 21). The operation begins when the Solar module harvests solar energy and delivers it to the charge controller. The controller regulates battery pack charging, ensuring a stable voltage supply and protecting against overcharge and deep discharge. The stored energy powers both the Control and Pump modules.

The system is managed by an ESP32-WROOM microcontroller, which executes programmed irrigation cycles and continuously monitors system variables. The input signals are received from a digital temperature sensor (DS18B20), which measures the internal conditions of the growth tower, and a water-level sensor, which monitors the availability of nutrient solution in the Reservoir module. These inputs enable basic fault detection (e.g., low reservoir level) and environmental tracking.

Irrigation is performed intermittently according to a predefined schedule. During each cycle, the microcontroller activates a relay that powers the pump, which draws nutrient solution from the reservoir and delivers it under pressure to the Filter module.

The nutrient solution passes through the mesh filter, removing suspended particles to prevent nozzle clogging. The filtered nutrient solution is then delivered to the vertical distribution pipe inside the Growth module. Nozzles atomize the nutrient solution as a fine mist, which is uniformly distributed across plant root zones. This process enhances oxygen availability while ensuring efficient nutrient uptake.

Excess nutrient solution drains by gravity back into the Reservoir module, maintaining a closed-loop system that minimizes water and fertilizer losses.

The ESP32 connects to a local router via Wi-Fi and transmits temperature and water-level data to a remote server over the Internet. The server stores the data and provides a web-based graphical user interface (GUI) for real-time monitoring and updating irrigation schedules. Historical performance data are logged in a database for review and analysis.

Continuous energy availability depends on solar irradiance and battery capacity. Proper filtration is critical to avoid nozzle obstruction. Irrigation frequency must be adjusted according to crop type and environmental conditions.

## 7 Validation and characterization

The performance of the TOTEM system was evaluated through a cultivation experiment using basil (*Ocimum basilicum*) as a model crop. Basil was selected for its rapid growth, sensitivity to water availability, and common use in CEA.

**Fig. 22:**
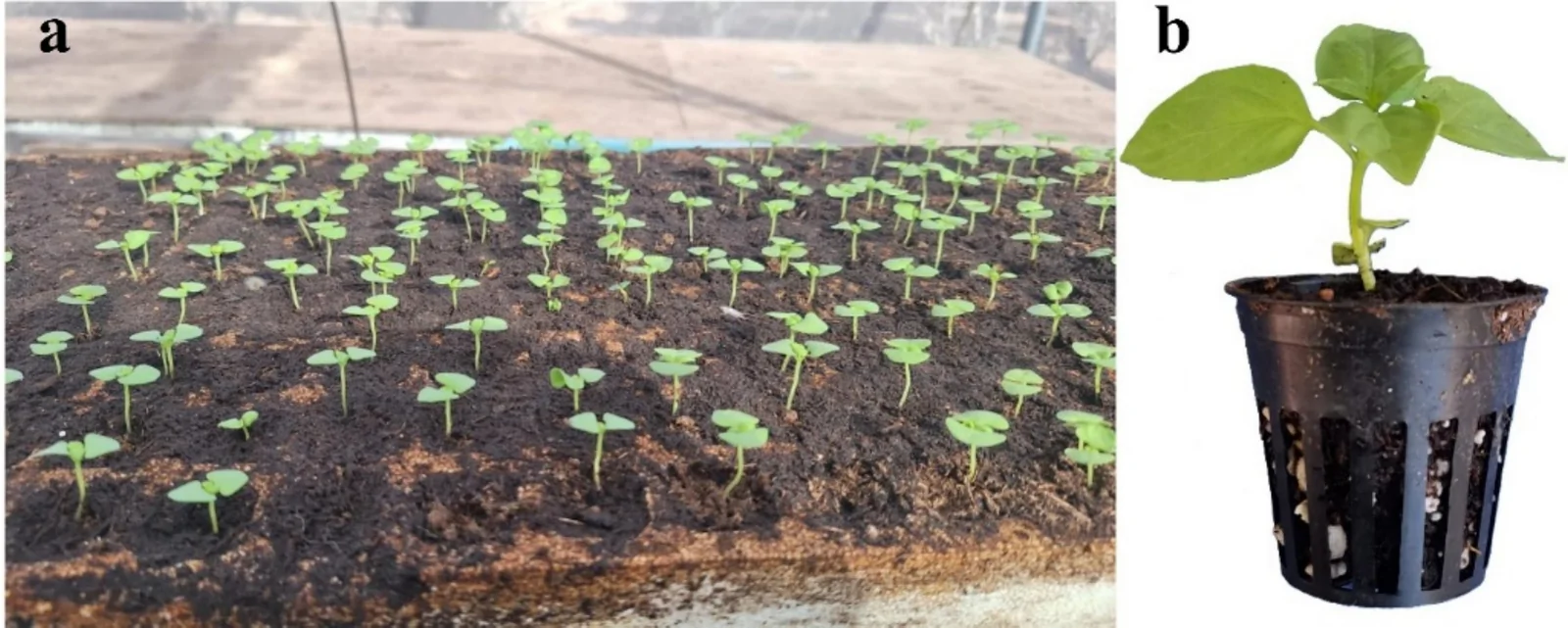
Basil cultivation: (a) seedling after germination and (b) transplanting into the plant cup.

Seedlings were transplanted into the TOTEM system after initial germination (Fig. 22). Plants were cultivated using a standard nutrient solution suitable for leafy crops. The irrigation was scheduled at 0.5 min ON and 29.5 min OFF to maintain root-zone moisture. A commercial balanced fertilizer (Triple 19; Nitrogen, Phosphorus, and Potassium, all at 19%, with micronutrients) was used (Table 13). The nutrient solution was prepared at a concentration of 1 g/l using fresh water with an electrical conductivity (EC) of 0.4 µs/cm and a pH of 6.13.

**Table 13:** Composition of the nutrient solution used for basil cultivation in the TOTEM system.

Element	Triple 19 (1 g/l ppm)
Nitrogen (N)	285.00
Phosphor (P)	285.00
Potassium (K)	285.00
Magnesium (Mg)	165.00
Iron (Fe)	0.60
Copper (Cu)	0.15
Zinc (Zn)	0.30
Manganese (Mn)	0.30
Boron (B)	0.15

**Fig. 23:**
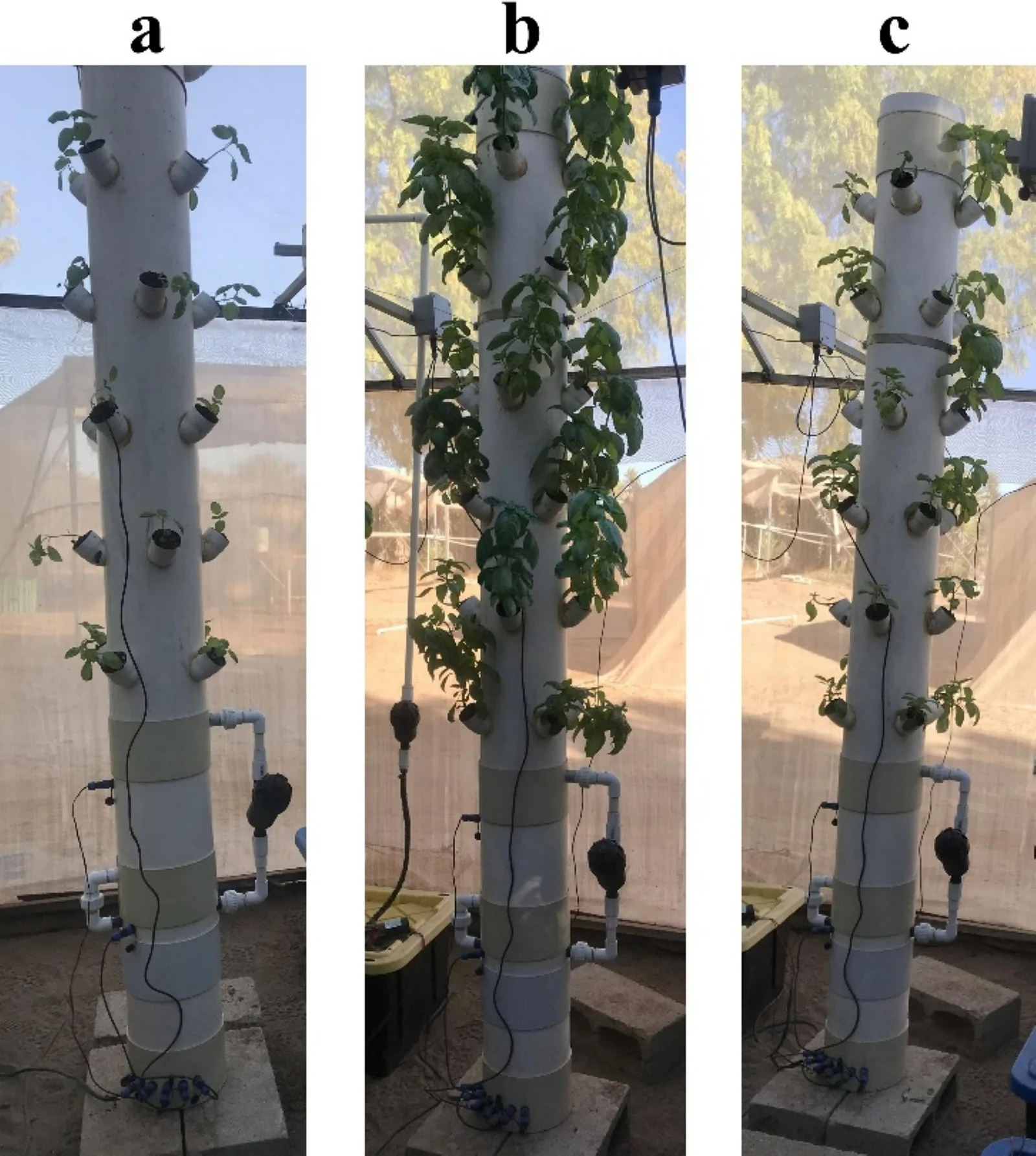
Basil plants in the TOTEM system: (a) initial stage after transplanting, (b) growth at 29 days, and (c) formative pruning at 30 days.

A total of 24 plants were grown simultaneously under shade greenhouse conditions, with ambient temperature, light, and humidity left uncontrolled. The experiment was conducted over a cultivation cycle of 55 days and included two sequential harvests, performed through leaf cutting following an initial formative pruning (Fig. 23).

**Fig. 24:**
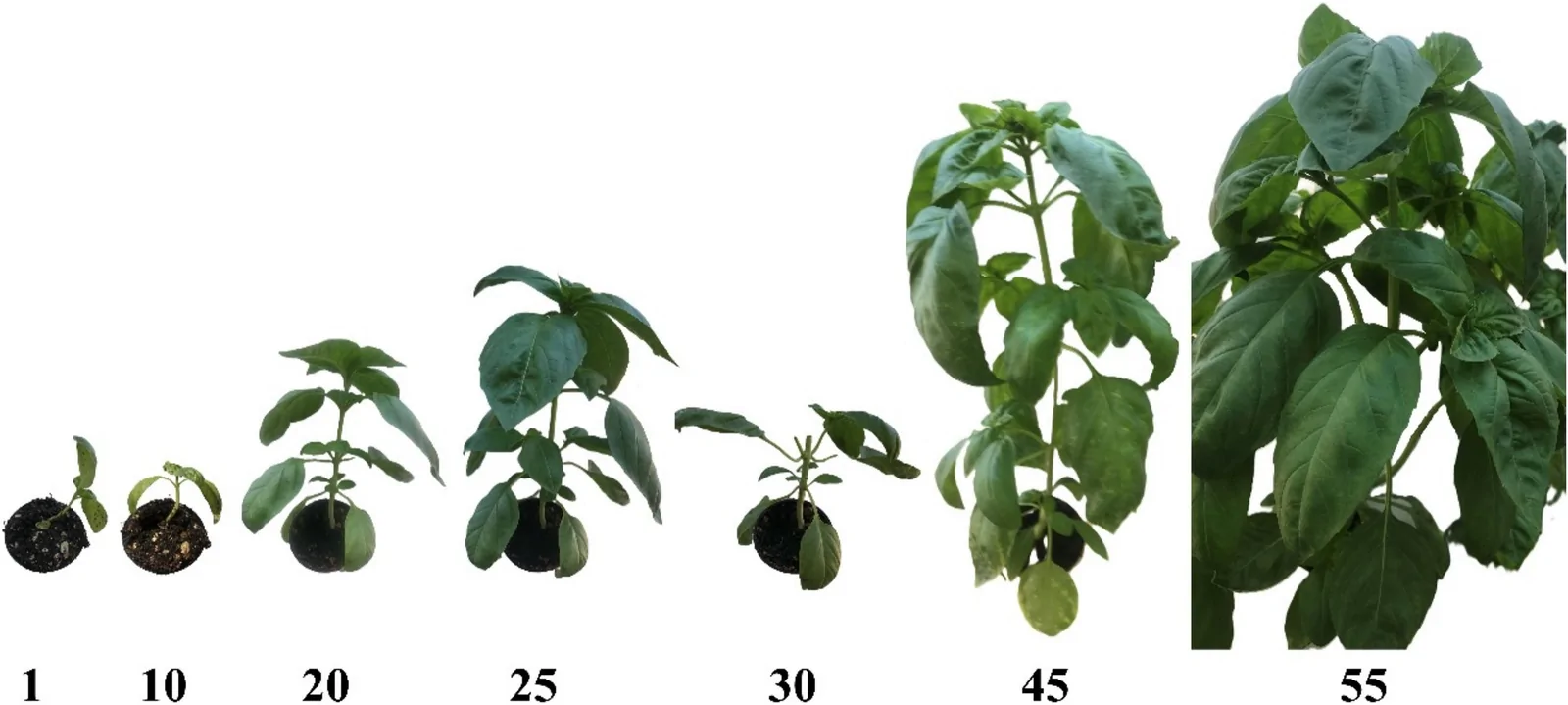
Growth progression of basil plants cultivated in the TOTEM system during the 55-day experimental period.

Formative pruning was performed 30 days after transplanting by removing the apical meristem to promote lateral branching and increase leaf production. The first harvest was conducted over 45 days by removing fully expanded leaves while maintaining sufficient foliage for continued growth. The second and final harvest was conducted on day 55 (Fig. 24).

Approximately 54 L of nutrient solution was used for the 24 basil plants during the 55-day cultivation period. Recirculation and recovery enabled repeated use of the solution, and periodic additions compensated for plant uptake and losses while maintaining the reservoir level. At the end of cultivation, the nutrient solution pH was 7.39 [36].

Growth and physiological parameters were measured at the final harvest (Table 14). The three sampled plants had a mean leaf area of 517.97 cm^2^, 31.33 leaves per plant, and a shoot length of 18.8 cm. Mean fresh and dry leaf weights were 11.97 and 1.26 g, respectively, corresponding to approximately 10.5% dry matter. Mean relative water content (RWC) was 82.18% [37]. Estimated nutrient-solution use was 0.041 l/plant per day.

**Table 14:** Growth and physiological parameters of basil plants in the TOTEM system 55 days after transplanting.

Variable	Sample 1	Sample 2	Sample 3	Mean
Leaf area (cm^2^)	589.53	414.15	550.24	**517.97**
Number of leaves	27	31	36	**31.33**
Shoot length (cm)	19.4	16	21	**18.8**
Fresh leaf weight (g)	13.4	9.5	13	**11.97**
Dry leaf weight (g)	1.4306	1.0534	1.2869	**1.26**
Fresh stem weight (g)	5.1	3.7	4.9	**4.57**
Dry stem weight (g)	0.423	0.3268	0.3891	**0.38**
RWC (%)	79.56	85.81	81.16	**82.18**

**Fig. 25:**
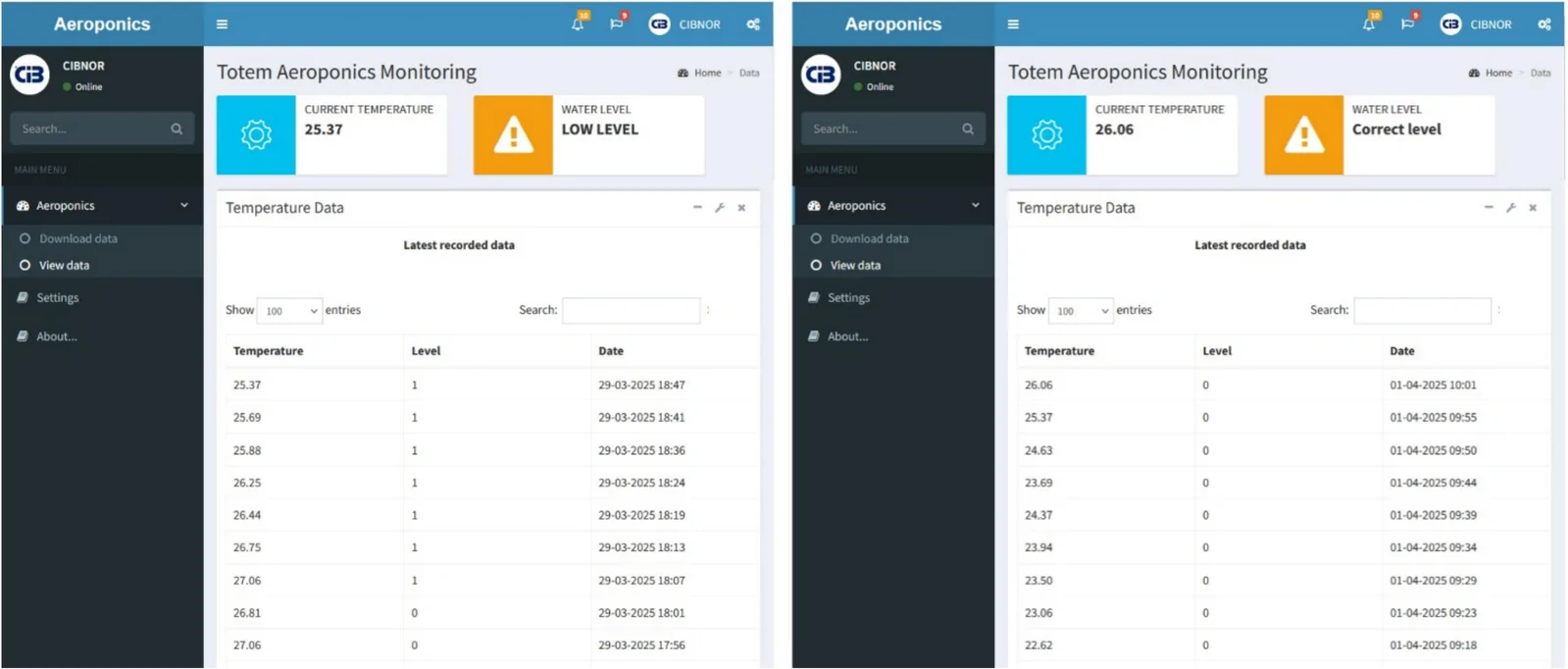
Real-time monitoring GUI displaying chamber temperature and nutrient solution level, showing a low-level alert (left) and a correct-level status (right).

**Fig. 26:**
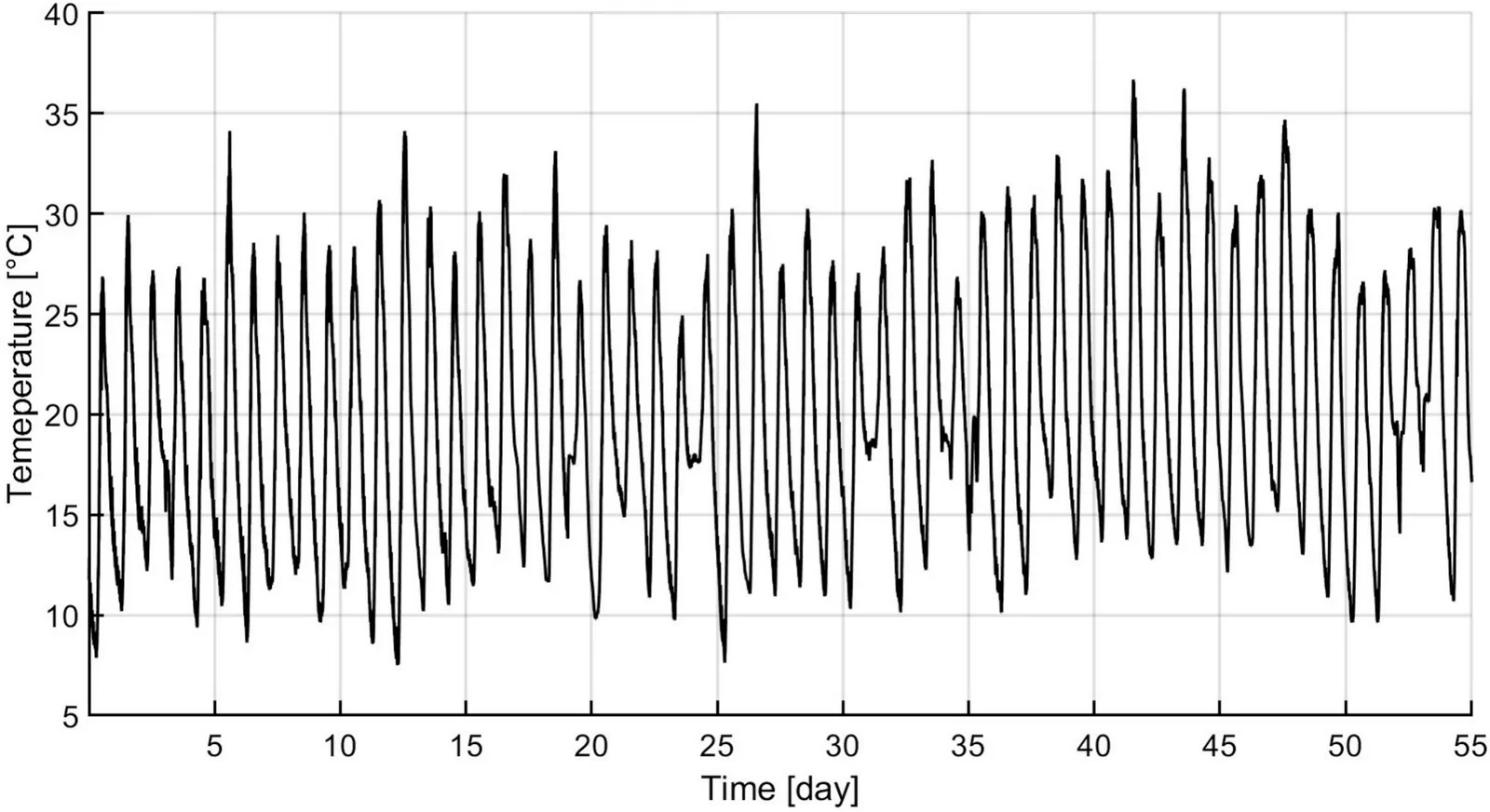
Chamber temperature profile over the 55-day cultivation cycle.

The real-time monitoring GUI (Fig. 25) displayed chamber temperature and reservoir level throughout the trial. Chamber temperature followed natural diurnal fluctuations from 14 to 38 °C during the 55-day cultivation cycle (Fig. 26).

The validation schedule comprised 0.5 min of pump operation followed by 29.5 min without irrigation, equivalent to 48 activation cycles per day. Based on pump ratings of 12 V and 4.0 A, the Pump module required 19.2 Wh/day. The ESP32-WROOM-32 microcontroller in the Control module operated continuously at 3.3 V; using a conservative active mode current of 0.24 A, its estimated demand was 19.0 Wh/day. Combined demand was therefore 38.2 Wh/day, or 44.9 Wh/day after applying a system efficiency of 0.85.

System sizing used a conservative solar resource of 3.9 peak sun hours per day for La Paz, Baja California Sur, corresponding to the lowest monthly climatological irradiation of 3.907 kWh/m^2^ per day in December. With a photovoltaic derating factor of 0.70, the 50 W solar panel (Solarever PLM-50P/12) provides an estimated 136.5 Wh/day, above the calculated minimum photovoltaic capacity of 16.5 W. The two parallel 12 V–12 Ah batteries provide 288 Wh nominally and 122.4 Wh of usable energy at a maximum depth of discharge of 50% and a battery efficiency of 85%, corresponding to approximately 2.7 days of autonomy without photovoltaic charging.

The TOTEM system remained operational throughout the 55-day basil cultivation period and completed the programmed irrigation schedule for one cultivation cycle.

### 7.1 Discussion

The basil trial demonstrated coordinated operation of irrigation control, monitoring, and solar energy supply under the tested shade-greenhouse conditions. Nutrient-solution recirculation and intermittent misting supported plant survival and two sequential harvests, while the reservoir-level and chamber-temperature sensors supplied operational data. The observed leaf development and mean RWC of 82.18% are consistent with maintained plant hydration during the trial, and the photovoltaic configuration provided the calculated capacity for off-grid operation. Collectively, these findings provide preliminary evidence of integrated prototype operation; comparative agronomic performance relative to commercial aeroponic systems or other cultivation methods requires controlled experiments.

The final nutrient-solution pH of 7.39 exceeded the mildly acidic range commonly targeted for soilless crop production. This deviation identifies a limitation in nutrient management and supports incorporating calibrated pH and EC sensors into a closed-loop control strategy to improve solution stability and crop uniformity.

The estimated nutrient-solution use of 0.041 l/plant per day was lower than the values reported for protected (approximately 0.6 l/plant per day) [38] and open-field systems (approximately 12.6 l/plant per day) [39]. Differences in climate, plant density, cultivation duration, system boundaries, and measurement methods restrict direct comparison, so the published values serve only as contextual references. Likewise, the three plants reported in Table 14 provide descriptive growth data rather than an estimate of variability across the 24 tower positions. The results therefore support an initial demonstration of prototype operation under the tested conditions.

The single-cycle evaluation characterizes operational and cultivation performance rather than long-term endurance, leaving system service life unresolved. Long-term reliability will depend primarily on hydraulic flow, structural integrity, control-system integrity, and energy-storage capacity. Preventive maintenance should include cleaning the mesh filter, misting nozzles, reservoir, and return lines to remove suspended solids and biofilm; inspecting tubing, seals, and waterproof connectors for leakage or deterioration; periodically verifying pump performance, sensor operation, battery condition, and charge-controller function; and inspecting outdoor PVC components for joint loosening and ultraviolet degradation.

Further research should use a matched commercial-tower comparator, randomized plant positions, adequate biological replication, repeated cultivation cycles, continuous water and energy metering, hydraulic monitoring, and records of maintenance and failures. Multi-cycle trials should record pump operating hours, flow rate or pressure, battery condition, failure events, maintenance time, and component replacements to quantify reliability and component durability. Evaluation across crop species and seasons, together with automated pH and EC management, would support broader assessment of scalability, life-cycle cost, and agronomic performance.

### 7.2 Conclusion

TOTEM integrates a 24-site vertical cultivation tower, recirculating low-pressure irrigation, mesh filtration, embedded scheduling and monitoring, and photovoltaic generation with battery storage in a modular open-hardware platform. During a 55-day basil cultivation period, the assembled prototype executed programmed irrigation and supported two sequential harvests under shade-greenhouse conditions. Its principal contribution is the documented integration of structural, hydraulic, electronic, software, and off-grid energy components, accompanied by design files and an itemized bill of materials. The experiment supports technical feasibility under the tested conditions, while comparative agronomic performance and long-term service life require controlled benchmarking and multi-cycle testing. Automated pH and EC management represents an additional priority for further development.

## Ethics statements

This study did not involve human subjects. No animal experiments were conducted in this research.

## Declaration of generative AI and AI-assisted technologies in the manuscript preparation process

During the preparation of this manuscript, the author(s) used Grammarly for checking grammar and spelling. The author(s) reviewed and edited the output as needed and take full responsibility for the content of the published article.

## CRediT authorship contribution statement

**Fernando D. Von Borstel:** Writing – original draft, Software, Methodology, Conceptualization. **Juan Francisco Villa-Medina:** Software, Resources, Investigation. **Alejandra Nieto-Garibay:** Validation, Formal analysis, Data curation. **Joaquín Gutiérrez:** Writing – review & editing, Supervision, Project administration.

## Funding

This research did not receive any specific grant from funding agencies in the public, commercial, or not-for-profit sectors.

## Declaration of competing interest

The authors declare that they have no known competing financial interests or personal relationships that could have appeared to influence the work reported in this paper.
